# Supplementary material for: Genome and single-cell RNA-sequencing of the earthworm Eisenia andrei identifies cellular mechanisms underlying regeneration
Source: Nat Commun. 2020 May 27;11:2656. doi: 10.1038/s41467-020-16454-8 (PMC7253469; doi:10.1038/s41467-020-16454-8)
Supplement: Supplementary file 1 — Supplementary Information [file 41467_2020_16454_MOESM1_ESM.pdf]

Supplementary Information

The Earthworm *Eisenia andrei* Genome and Single-cell RNA-Sequencing Reveal Molecular and Cellular Mechanisms underlying Regeneration

Shao, Wang, Zhang, Li et al.

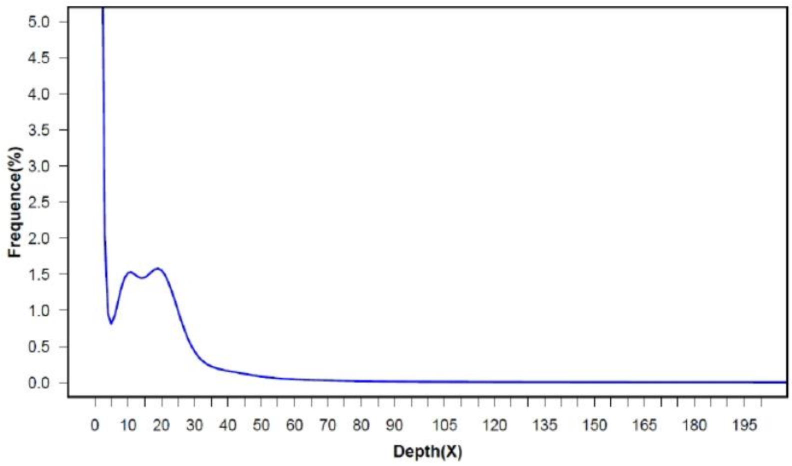

Supplementary Figure 1 | Genome size of the earthworm estimated from 17 k-mers using 34.7Gb next generation sequencing reads. In detail, K-mer=17, kmer num=25,536,306,648, k-mer depth=20, genome size=1,276,815,332bp and coverage (X)=24.

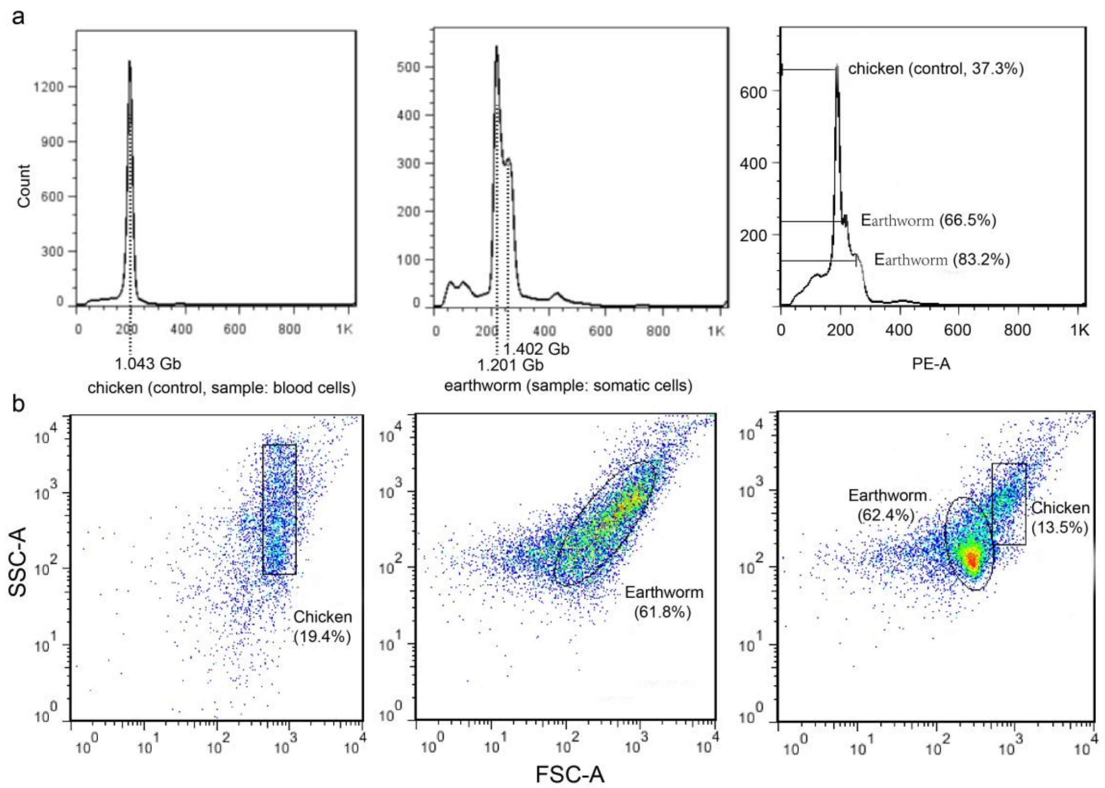

Supplementary Figure 2 | Evaluation of genome size for the earthworm using flow cytometry. The different data plotting styles were showed to measure genome size of earthworm. **a,** Flow cytometry histograms of the earthworm somatic cell samples and chicken blood cells obtained by PI fluorescence dye excitation and counts representing the cell population. **b,** Flow cytometry scatter plots. The gating strategy was decided by the empirical balance between relative size of the cells and the granularity. The domesticated chicken was utilized as a control with the known genome size (*Gallus gallus*, GRCg6a: 1.043 Gb).

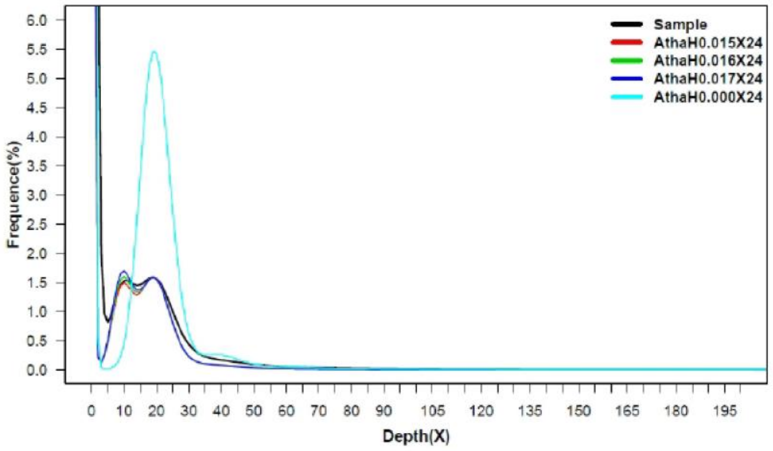

Supplementary Figure 3 | Simulated heterozygosity curve for earthworm. The x axis shows k-mer depth (X), and y axis exhibits k-mer depth frequency. The AthaH0.015X24 simulation shows the 1.5% Atha-depth heterozygosity (H) (X is 24). The earthworm heterozygosity is identified as 1.5%.

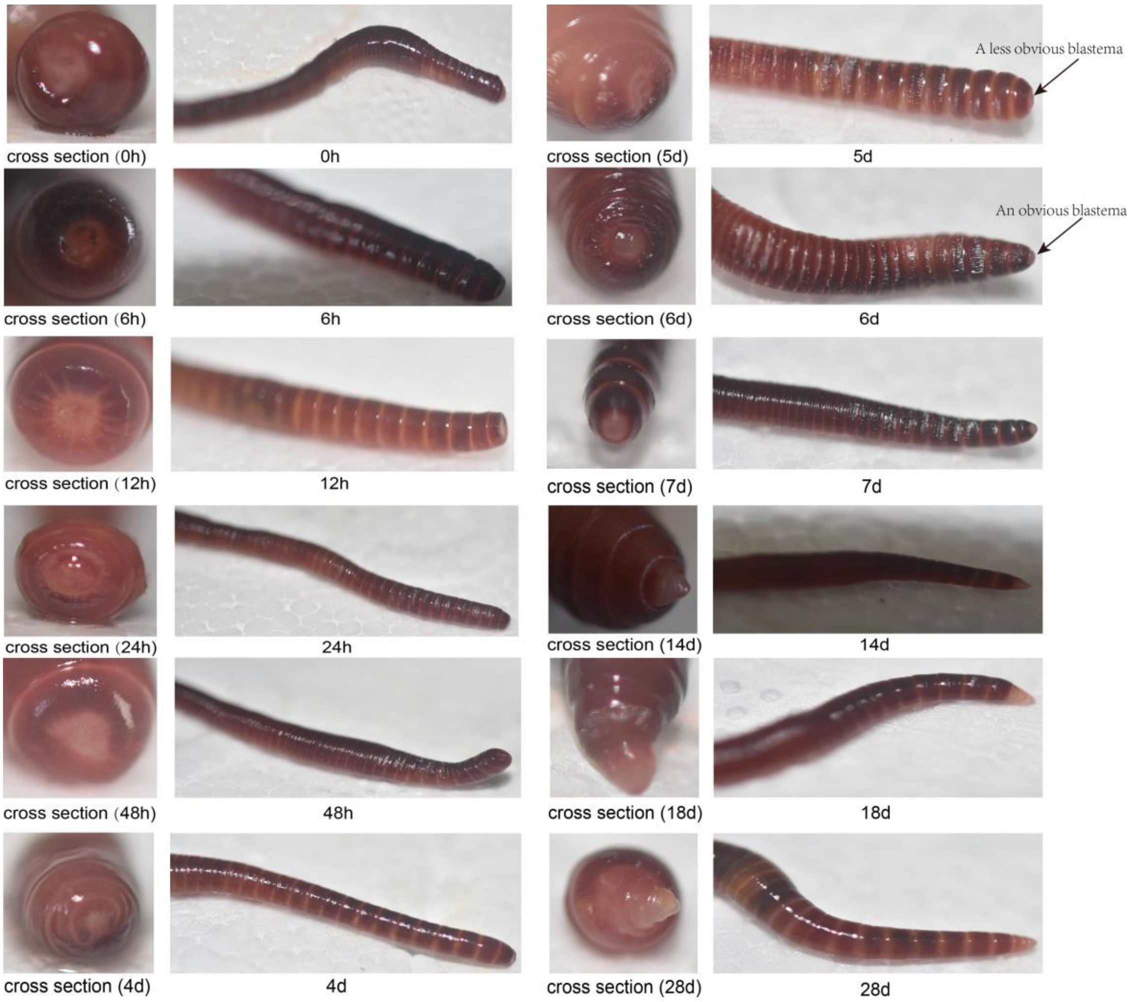

Supplementary Figure 4 | Snapshots of cross sections at 12 regenerative time points after post-amputation. At 5 days post-amputation a small blastema was formed, and at 6 days post-amputation an obvious blastema was observed.

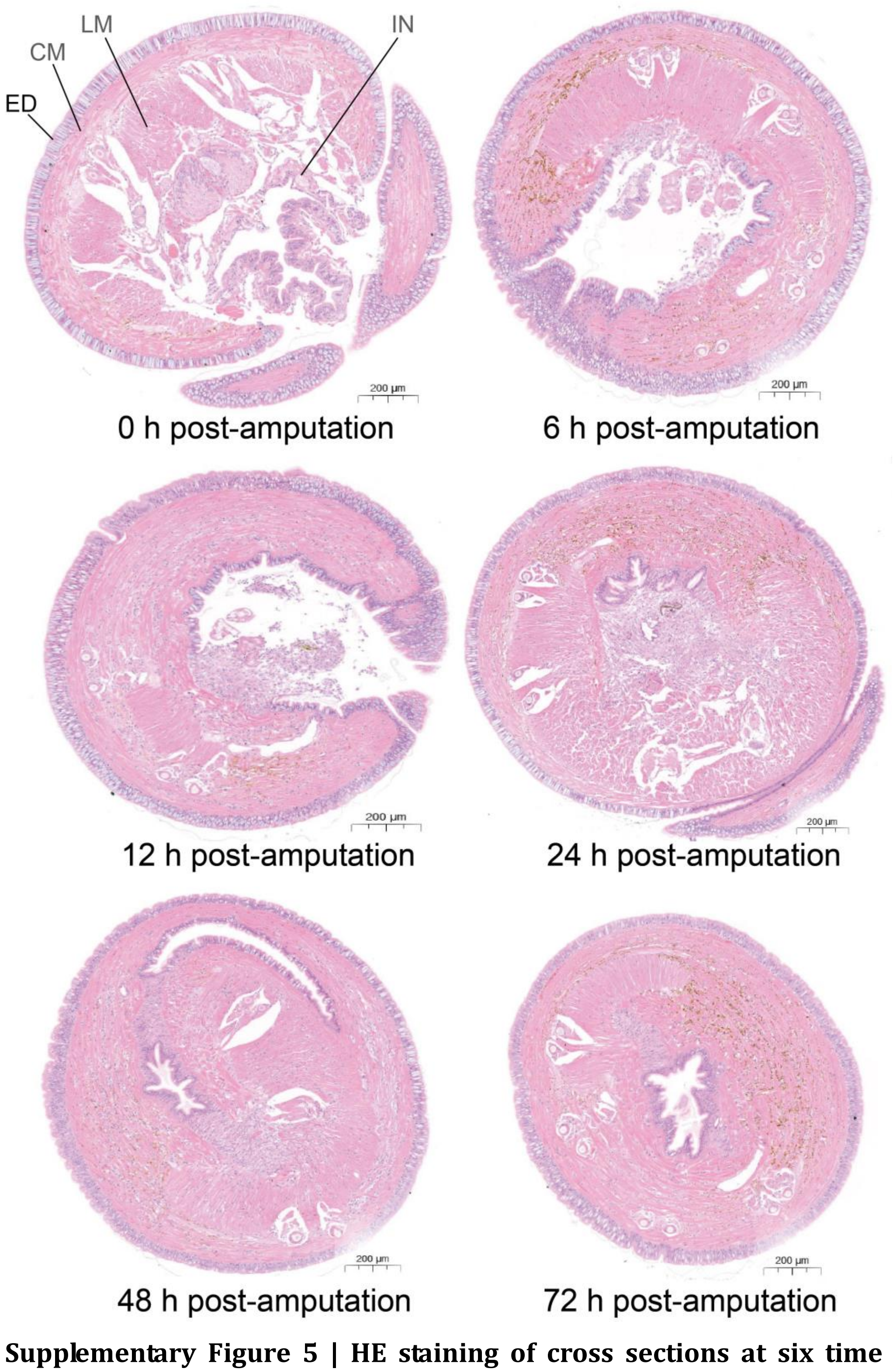

Supplementary Figure 5 | HE staining of cross sections at six time points post-amputation. The six time points included 0, 6, 12, 24, 48 and 72 hours post-amputation. The scale size was 200 μm. The different structure layers were labeled. ED: epidermis; CM: circular muscle; LM: longitudinal muscle; IN: intestine. Similar results could be repeated by three independently biological experiments.

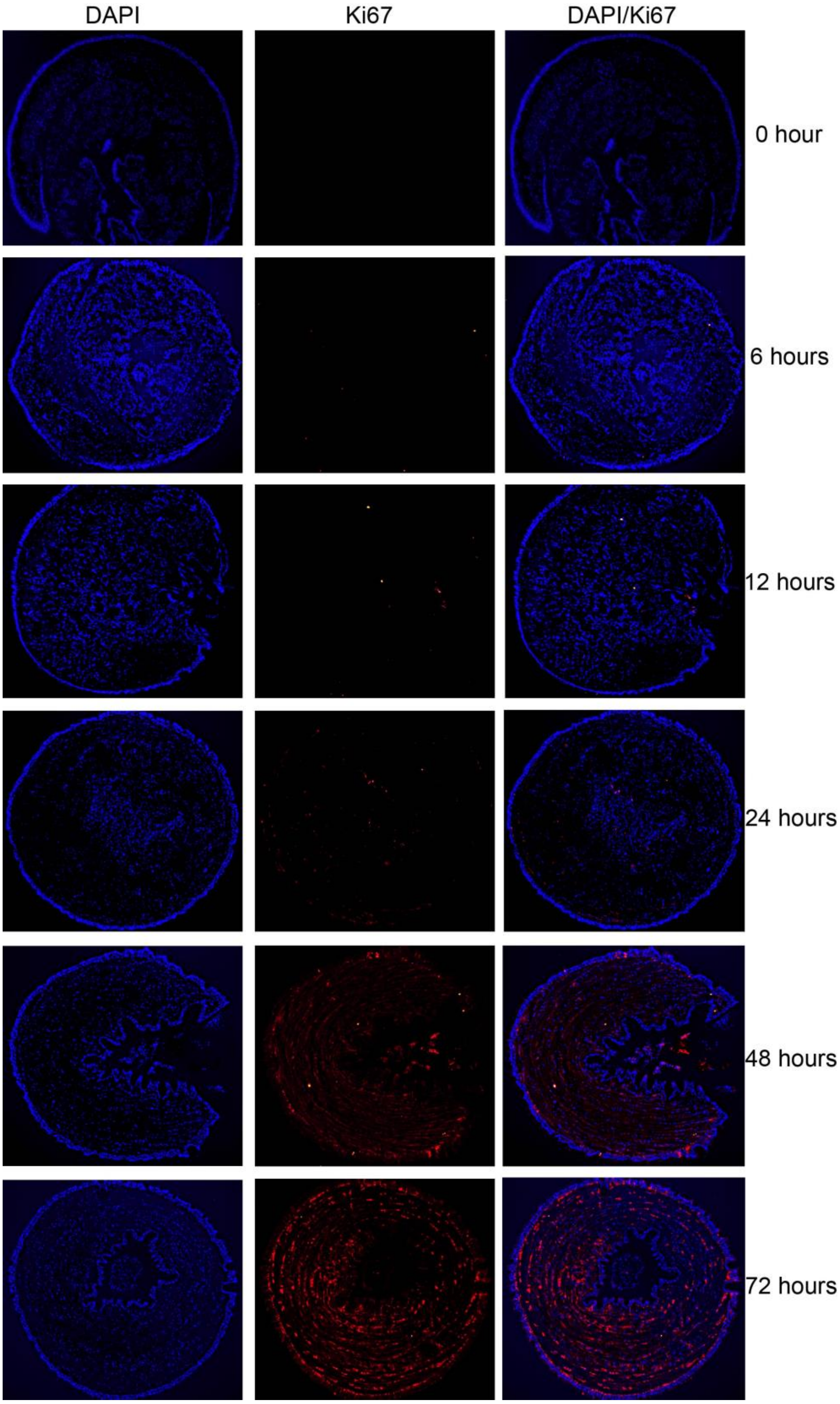

Supplementary Figure 6 | Cell proliferation experiments using a marker Ki-67 by Immunofluorescent double staining at 0, 6 12, 24, 48 and 72 hours post-amputation. And the red fluorescence represented signals and the blue fluorescence represented cell nucleus. Similar results could be obtained under two independently biological experiments.

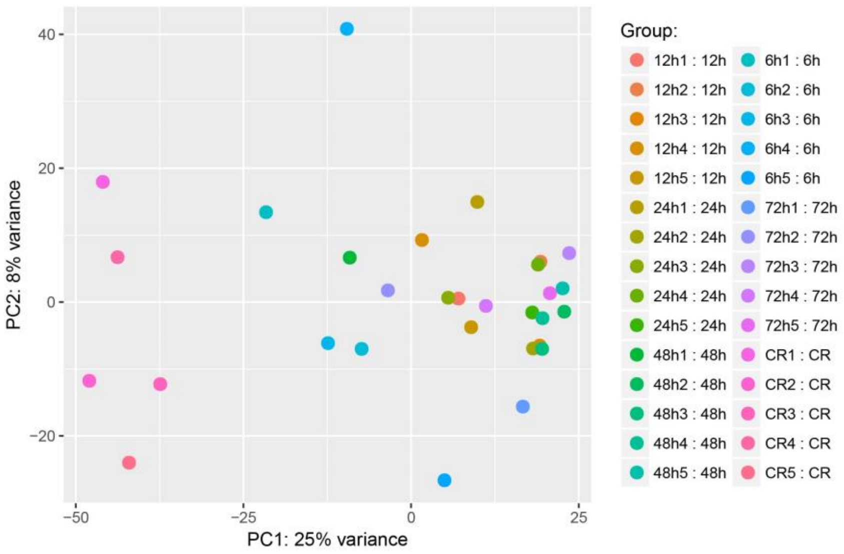

Supplementary Figure 7 | Expression profile PCA plot of 30 transcriptomes with 6 regeneration time-points. Controls (before regeneration) and experimental samples (regeneration) can be clearly split by PC1 (25% variance).

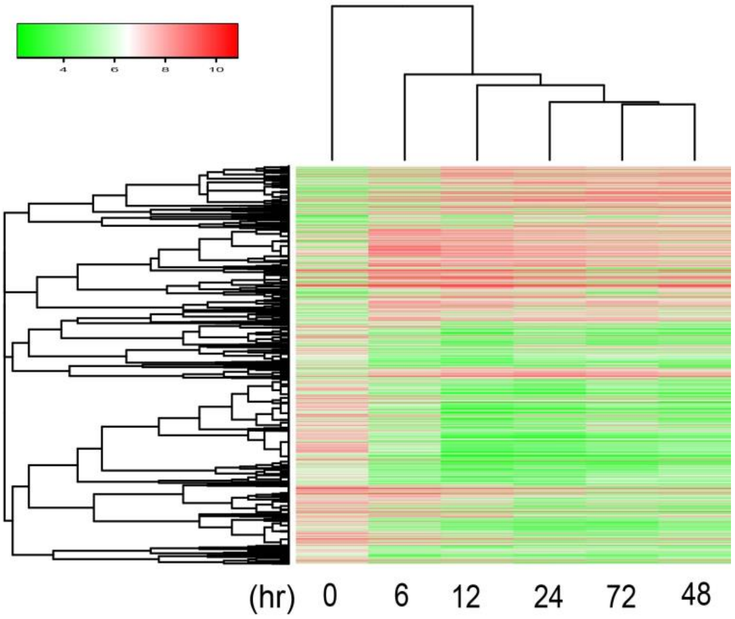

Supplementary Figure 8 | Heatmap of 6048 DEGs with their changed expression profiles at least in one regeneration time point. This expression profile represented a whole expression mode of DEGs during the regenerative early stages of earthworms. The expression profile of DEGs was split by regenerative time-order. 6048 DEGs were originated from comparing control stage to all other time points. The expression profile heatmap by using all of DEGs for each stage including control stage (0 hour) was plotted to show the whole expression pattern. The red color in the legend represented higher expression level and the green color represented lower gene expression level.

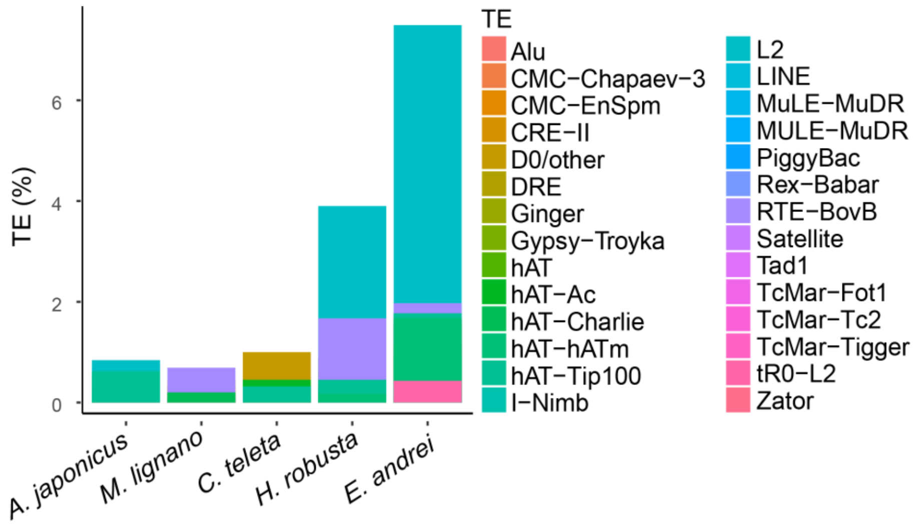

Supplementary Figure 9 | Content of representative TEs in the genomes of different invertebrates. 28 representative TEs were compared among five species including *E. Andrei*, *A. japonicas*, *M. lignano*, *C. teleta*, and *H. robusta*. Y-axis represented the % content of TEs in different species genomes, and X-axis represented different species.

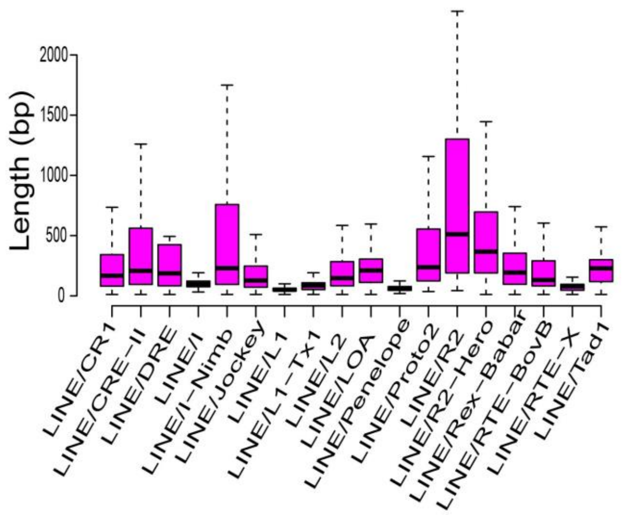

Supplementary Figure 10 | Boxplot of length distribution for the LINE family in earthworm. All of LINE family members were showed by X-axis. For each LINE TE, the distribution of the length was plotted. The sample sizes of LINE subclass were showed in the following: CR1 (n=37,125), CRE-Ⅱ(n=36,513), DRE (n=3,201), I (n=1,531), I-Nimb (n=5,585), Jockey (n=1,698), L1 (n=1,177), L1-Tx1 (n=4,174), L2 (n=457,629), LOA (n=2,262), Penelope (n=179), Proto2 (n=405), R2 (n=240), R2-Hero (n=2206), Rex-Babar (n=13,200), RTE-BovB (n=115,985), RTE-X (n=2,007) and Tad1 (n=6,691). In boxplot, central black thick lines represented medians; boxes represented interquartile ranges including 25th percentile (Q1) and 75th percentile (Q3); thin black lines from up to down illustrated maximum and minimum values, respectively; and whiskers showed 95% confidence intervals.

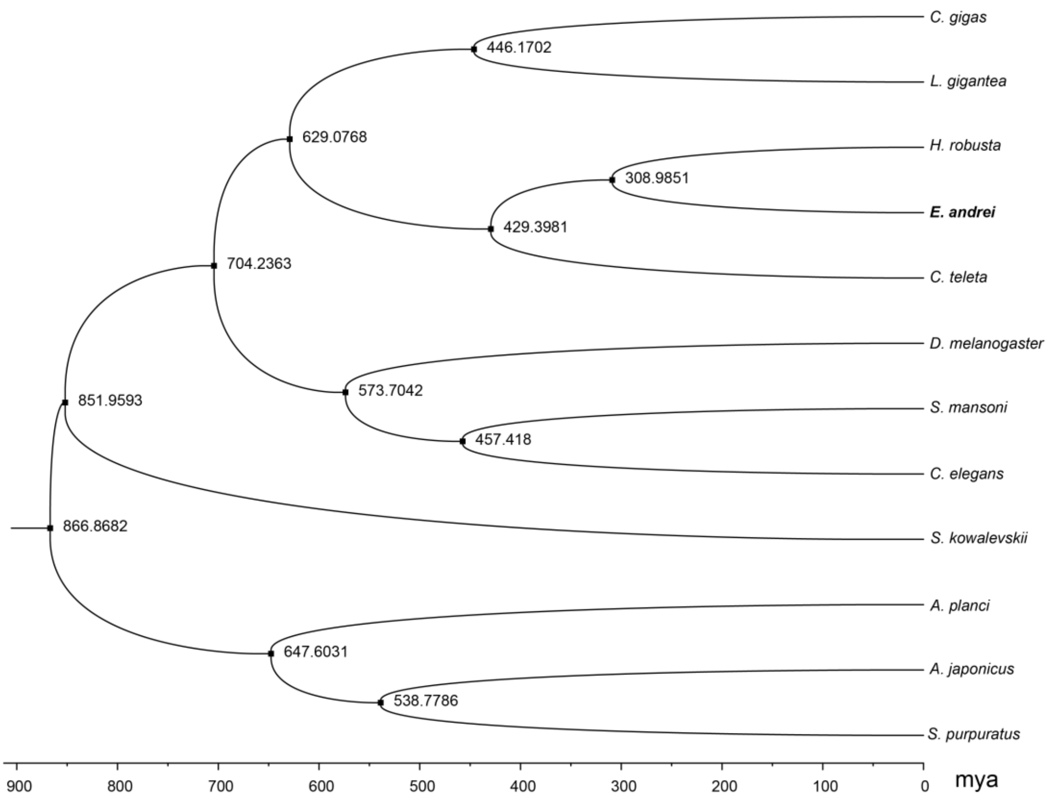

Supplementary Figure 11 | A time-scale species tree of invertebrates. The phylogeny was based concatenated one-to-orthologous genes using RAxML with PROTGAMMAAUTO model and 100 bootstraps. MCMCtree in PAML packages was utilized to estimate divergence time. Fossil calibration points were obtained from TimeTree (http://www.timetree.org/). All of nodal ages were marked.

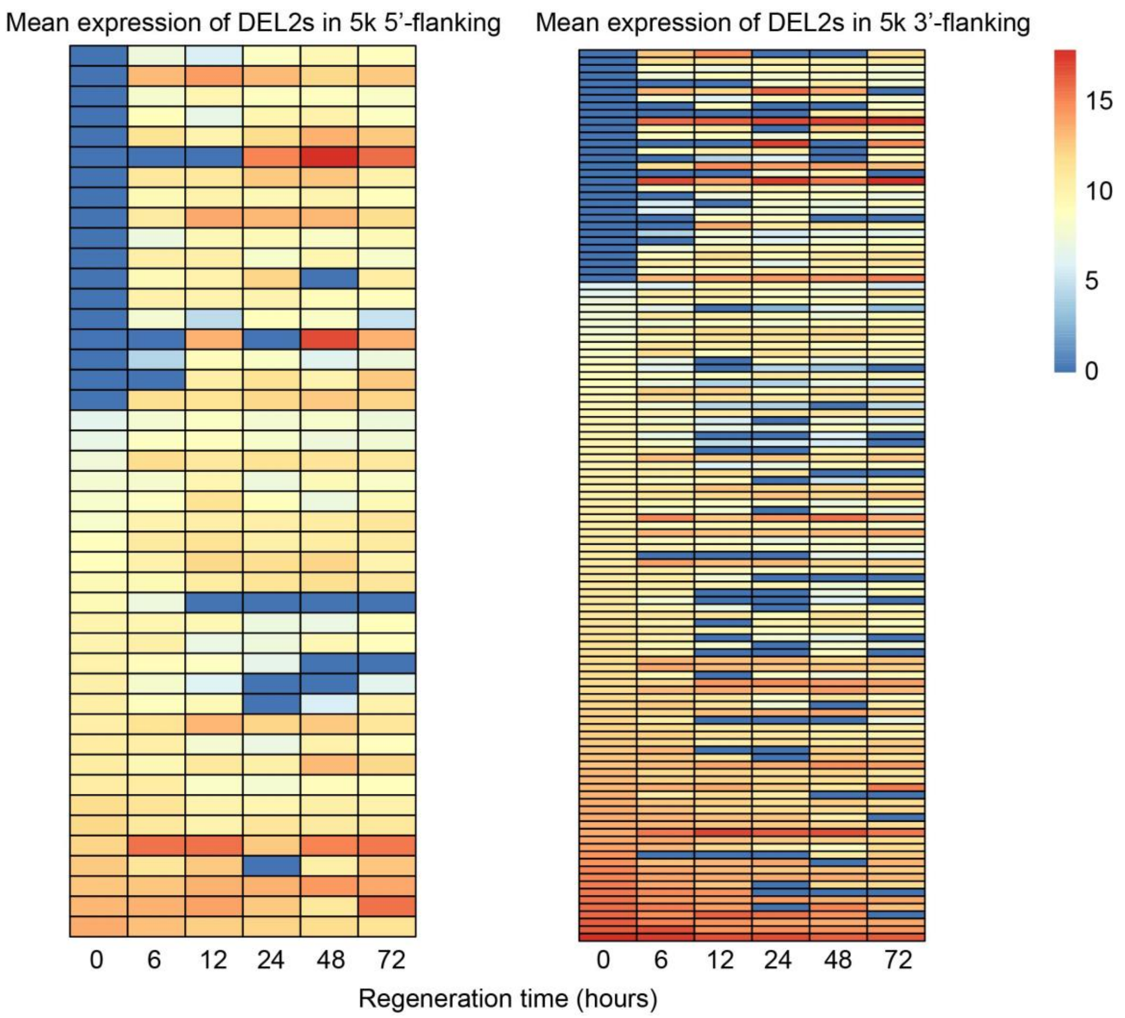

Supplementary Figure 12 | Expression profiles of differentially expressed LINE2 elements in 5k 5-flanking and 5k 3-flanking of coding genes during regeneration process. DEL2 represented differentially expressed LINE2 elements. Dark orange represented higher expression levels, and dark blue represented lower expression levels.

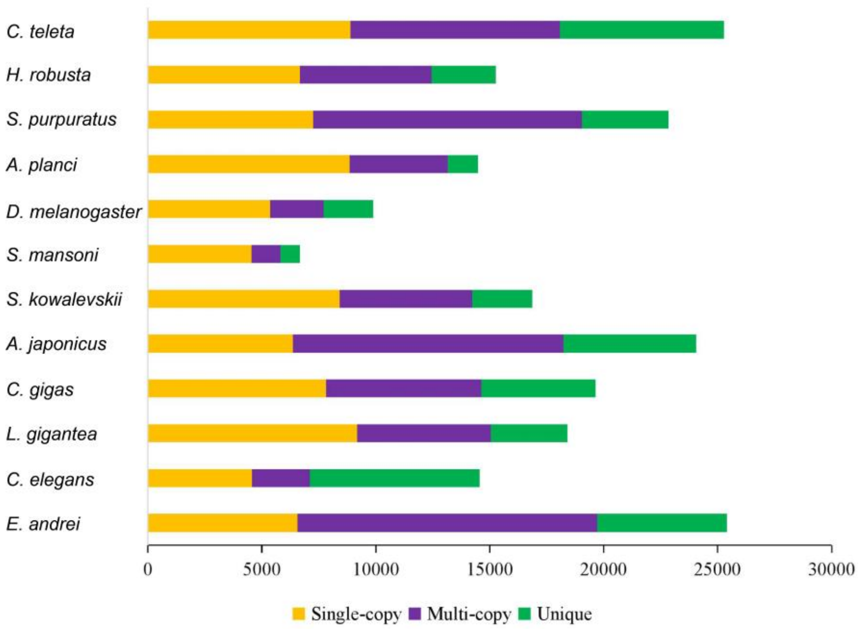

Supplementary Figure 13 | Comparison of the gene repertoires of 12 invertebrate genomes. Single-copy orthologues, multi-copy orthologues and unique genes across 12 species are shown by different colors (orange, blue and green).

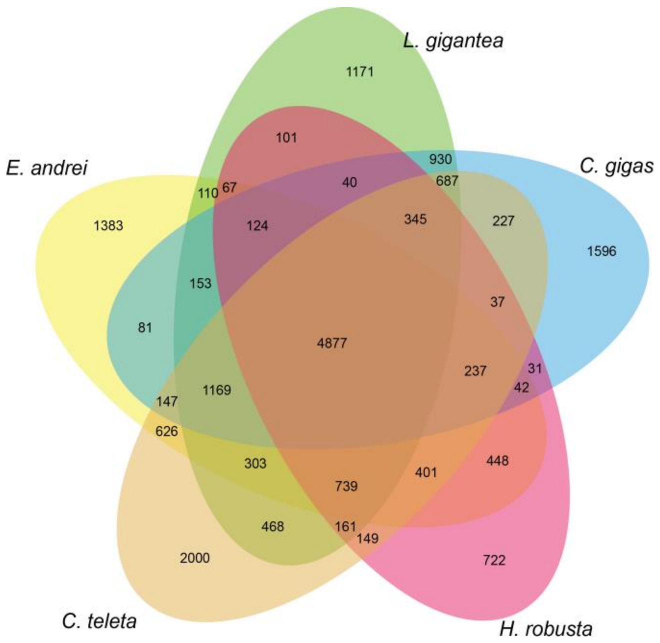

Supplementary Figure 14 | Co-shared gene families in the genomes of five closely related species. Five representative species include *E. andrei*, *H. robusta*, *C. teleta*, *C. gigas*, and *L. gigantea*.

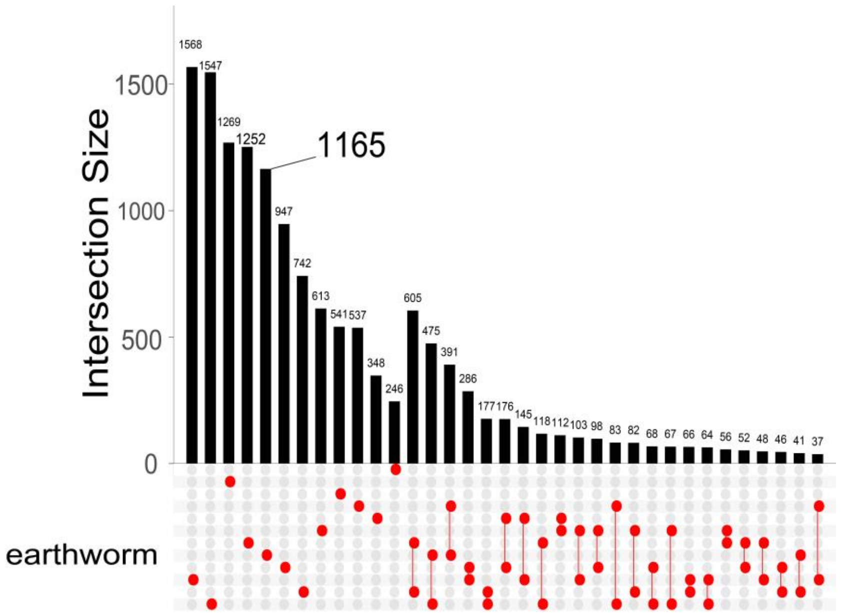

Supplementary Figure 15 | Intersection size analysis of gene family catalogues across 12 invertebrates. Parts of the intersections are shown, and the unique gene families of the earthworm are highlighted.

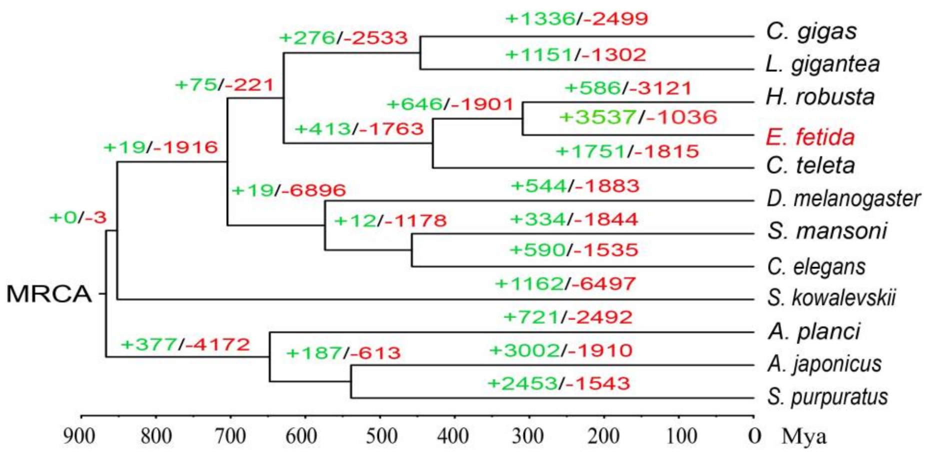

Supplementary Figure 16 | Evolution of gene families in the earthworm *E. fetida* genome. a, Expansion/constraction of gene families for 12 invertebates. Expanded gene families are shown in green and contracted gene families in red at the whole genome levels. The *E. fetida* branch was highlighted by red.

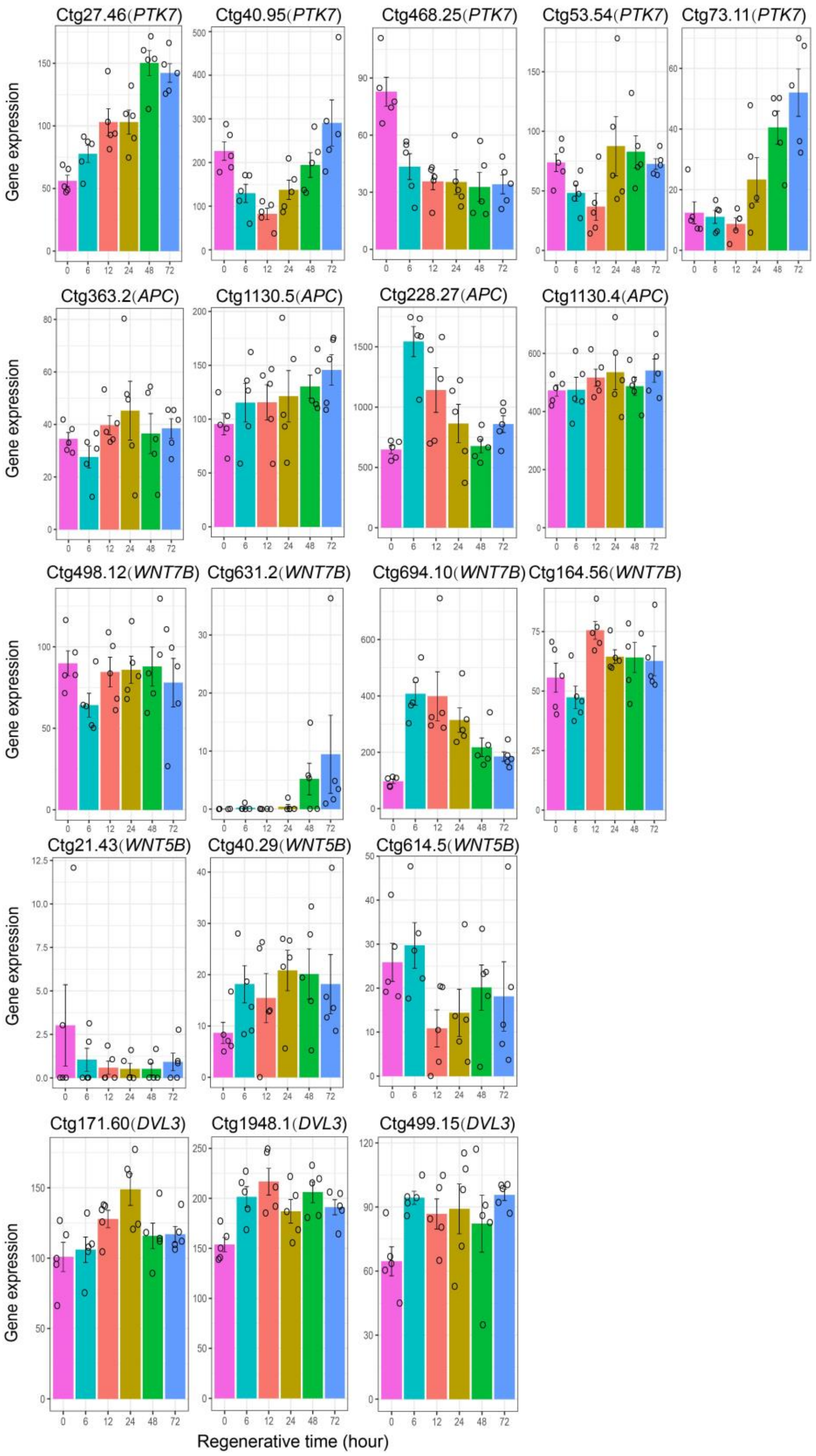

Supplementary Figure 17 | Expression levels of representative gene family members in WNT signal pathway. X-axis represents regenerative time points (hour). Each stage included 5 biological replicates (n=5). The error bars were calculated by using standard error of the mean (s.e.m).

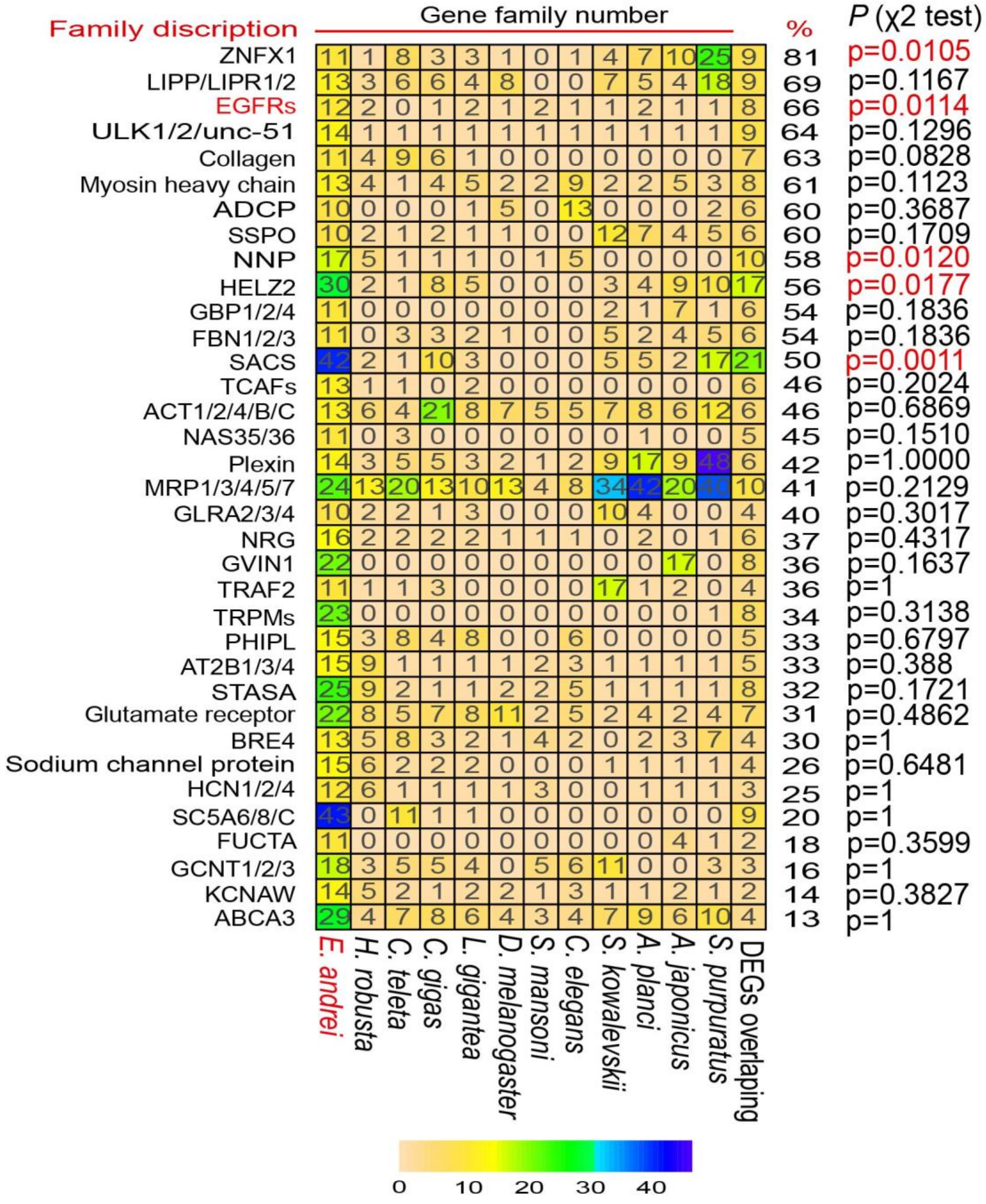

Supplementary Figure 18 | A heatmap graph of 35 gene families originated from 186 significantly expanded gene families in the earthworm branch with over 10% of their family members displaying significant expression changes during regeneration. The darker color indicates more family members. The last column number of the heatmap showed the number of co-shared genes between members of an assigned gene family and all of DEGs during early stages of regeneration. For each candidate gene family, a random test was done between observed value of DEGs in regeneration and randomly produced value by using X^2 test with Yates' continuity correction.

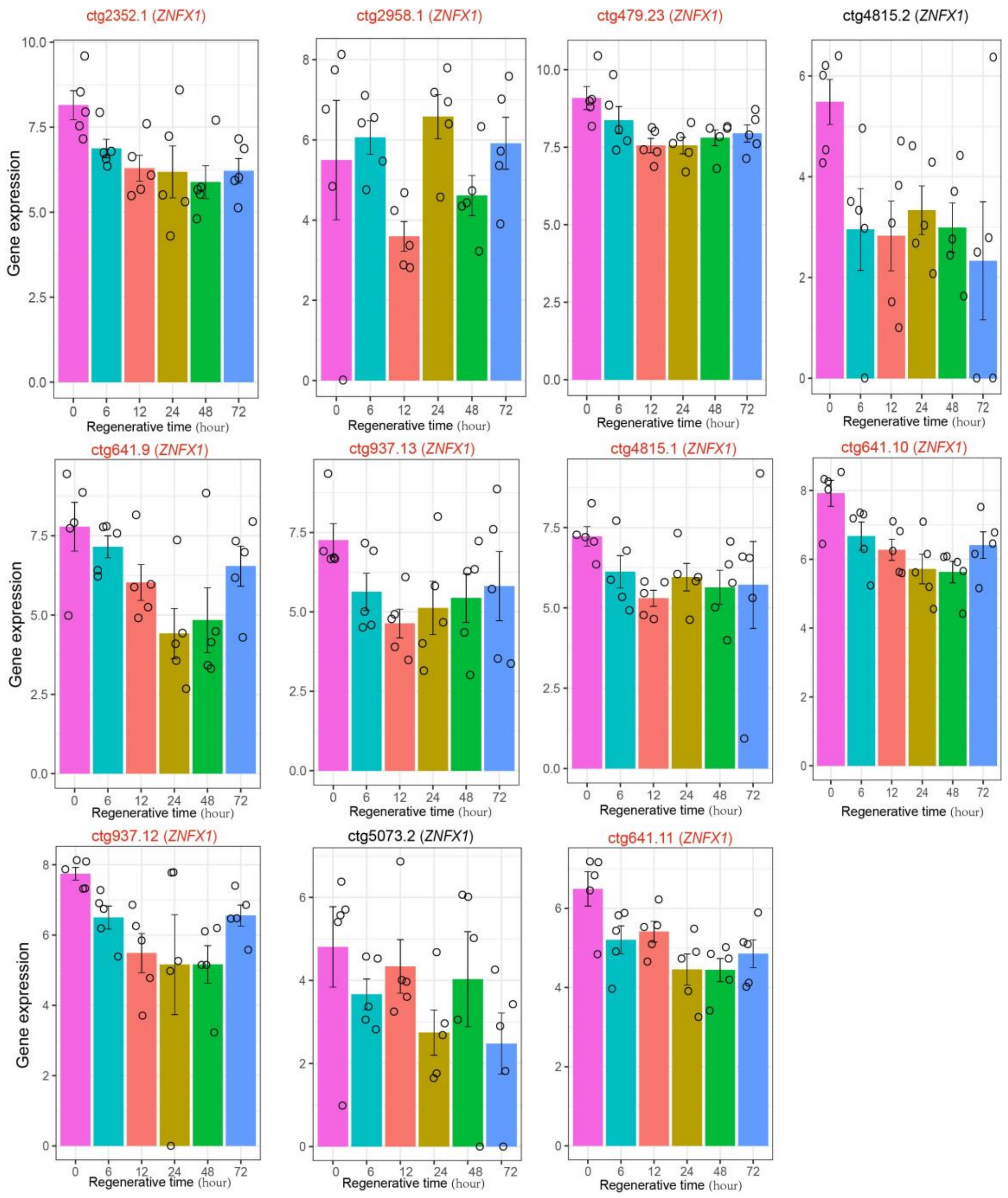

Supplementary Figure 19 | Differentially expressed analyses of *ZNFX1* copies during regenerative processes in the earthworm using different time points post-amputation. The significant levels were decided by cuffdiff algorithm in Cufflinks and FDR Benjamini-Hochberg multiple correction were performed (Adjusted P value<0.05). The DEGs were highlighted by red. For each regenerative stage, 5 biological replicates for each stage per gene were utilized (n=5). The error bars were calculated by using s.e.m. The adjusted p value for each gene were provided as follows: ctg2352.1: P=7.39985e-04 (0 vs. 6), P=5.60735e-04 (0 vs. 12), P=4.24259e-03 (0 vs. 24), P= 7.0794e-04 (0 vs. 48), P= 6.25935e-04 (0 vs. 72); ctg2958.1: P=0.224247 (0 vs. 6), P=5.60735e-04 (0 vs. 12), P=0.954624 (0 vs. 24), P=2.37766e-06 (0 vs. 48), P= 0.330401 (0 vs. 72); ctg479.23: P=0.137848 (0 vs. 6), P=5.60735e-04 (0 vs. 12), P= 5.89051e-04 (0 vs. 24), P=7.0794e-04 (0

vs. 48), P=6.25935e-04 (0 vs. 72); ctg4815.2: P=1 (0 vs. 6), P=1 (0 vs. 12), P=1 (0 vs. 24), P=1 (0 vs. 48), P=1 (0 vs. 72); ctg641.9: P=9.51733e-03 (0 vs. 6), P=5.60735e-04 (0 vs. 12), P=5.89051e-04 (0 vs. 24), P=0.0105512 (0 vs. 48), P= 0.00116472 (0 vs. 72); ctg937.13: P=7.39985e-04 (0 vs. 6), P=5.60735e-04 (0 vs. 12), P=5.85558e-03 (0 vs. 24), P=1.30999e-03 (0 vs. 48), P= 0.379193 (0 vs. 72); ctg4815.1: P=0.0806205 (0 vs. 6), P= 5.60735e-04 (0 vs. 12), P=0.0132856 (0 vs. 24), P=5.45699e-03 (0 vs. 48), P=0.981583 (0 vs. 72); ctg641.10: P=1.91737e-03 (0 vs. 6), P=5.60735e-04 (0 vs. 12), P=5.89051e-04 (0 vs. 24), P=7.0794e-04 (0 vs. 48), P=6.25935e-04 (0 vs. 72); ctg937.12: P=4.80706e-03 (0 vs. 6), P=5.60735e-04 (0 vs. 12), P=0.0201133 (0 vs. 24), P=7.0794e-04 (0 vs. 48), P=7.25809e-03 (0 vs. 72); ctg5073.2: P=1 (0 vs. 6), P=1 (0 vs. 12), P=1 (0 vs. 24), P=1 (0 vs. 48), P=1 (0 vs. 72); ctg641.11: P= 0.0188427 (0 vs. 6), P= 0.0207513 (0 vs. 12), P=1.97253e-03 (0 vs. 24), P=7.0794e-04 (0 vs. 48), P=2.0991e-03 (0 vs. 72).

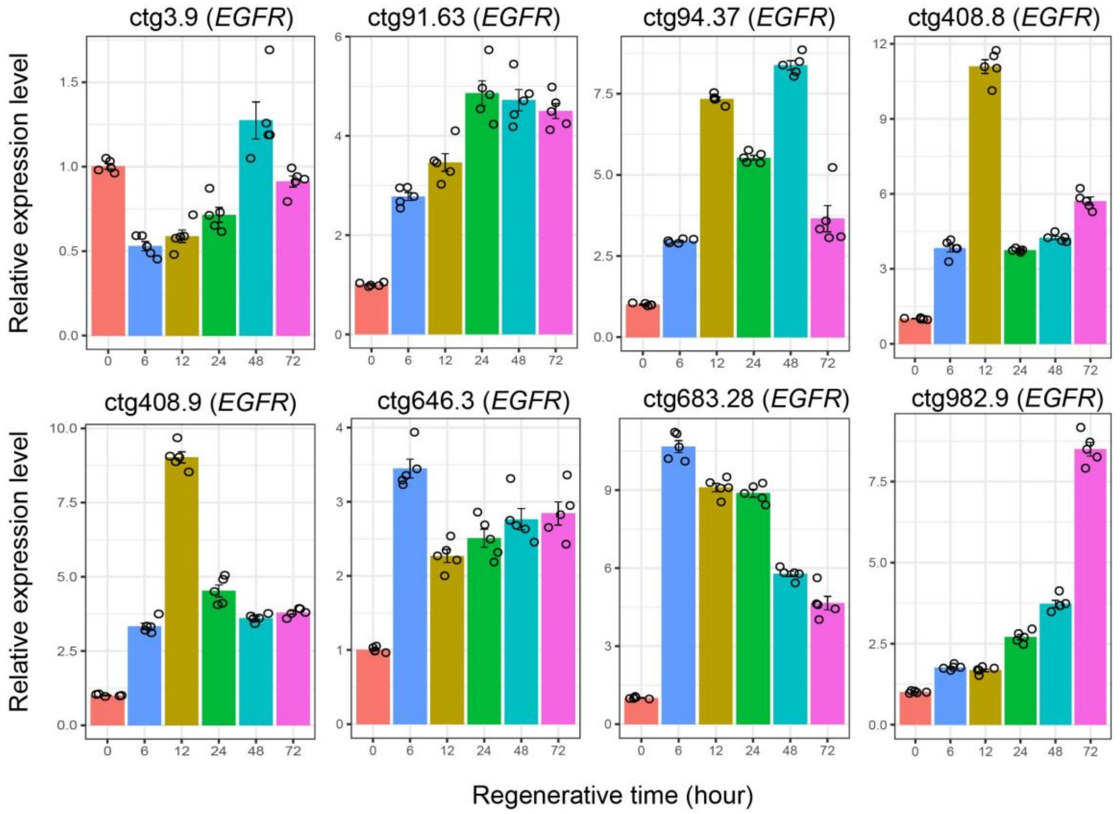

Supplementary Figure 20 | qPCR analyses of *EGFR* copies during regenerative processes in the earthworm using different time points post-amputation. Five biological replicates for each time point (n=5) were used and β-actin was served as a reference gene to normalize the relative mRNA expression levels. The error bars were calculated by using the s.e.m. The significance levels between control (regenerative 0 hour) and other stages were calculated by unpaired two tailed t-test. The p values were as follows: ctg3.9: p=0.004731075 (0 vs. 6), p=0.057366 (0 vs. 12), p=0.040812 (0 vs 24), p=0.394488 (0 vs. 48) and p=0.393905 (0 vs. 72); ctg91.63: p=0.002247 (0 vs. 6), p=0.003153 (0 vs. 12), p=0.001503 (0 vs. 24), p=0.001081 (0 vs. 48) and p=0.001573 (0 vs. 72); ctg94.37: p=0.00011 (0 vs. 6); p=1.12e-05 (0 vs. 12), p=0.00145 (0 vs. 24), p=2.13e-05 (0 vs. 48) and p=0.06674 (0 vs 72); ctg408.8: p=0.001907 (0 vs. 6), p=0.000103 (0 vs. 12), p=7.65e-05 (0 vs. 24), p=5.67e-05 (0 vs. 48) and p=0.00047 (0 vs. 72); ctg408.9: p=0.001355 (0 vs. 6), p=2.12e-05 (0 vs.12), p=0.001512 (0 vs. 24), p=0.000184 (0 vs. 48), p=0.000128 (0 vs. 72); ctg646.3: p=0.001388 (0 vs. 6), p=0.003862 (0 vs. 12), p=0.009538 (0 vs. 24), p=0.005377 (0 vs. 48) and p=0.004623 (0 vs. 72); ctg683.28: p=3.16e-05 (0 vs. 6), p=4e-05 (0 vs. 12), p=1.58e-05 (0 vs. 24), p=8.9e-05 (0 vs. 48) and p=0.00212 (0 vs. 72); ctg982.9: p=0.001504 (0 vs. 6), p=0.004977 (0 vs. 12), p=0.001851 (0 vs. 24), p=0.001293 (0 vs. 48) and p=0.000111(0 vs. 72).

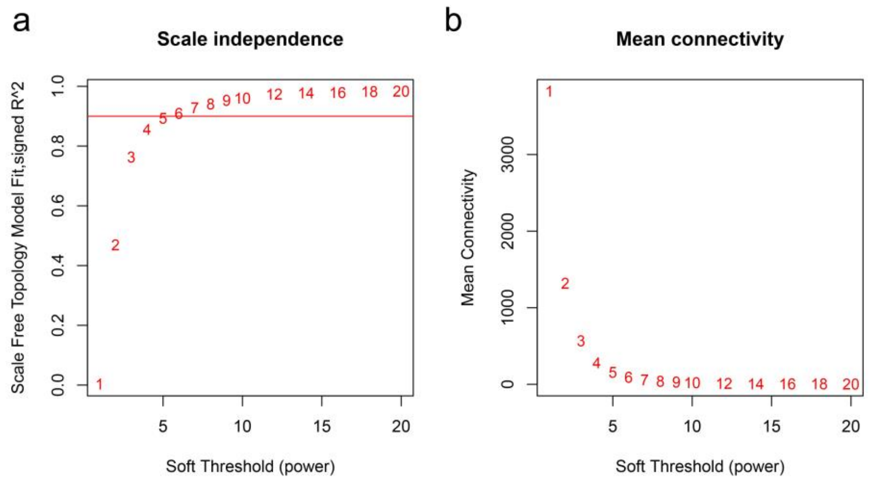

Supplementary Figure 21 | Analysis of the network topology for various soft-thresholding powers. The powers vs. scale independence and mean connectivity were showed. **a**, Panel shows the scale-free fit index (y-axis) as a function of the soft-thresholding power (x-axis). The lowest power 6 for the scale-free topology fit reached 0.90. When power equals to 10, then the scale-free topology fit was saturated. **b**, Panel displays the mean connectivity (degree, y-axis) as a function of the soft-thresholding power (x-axis).

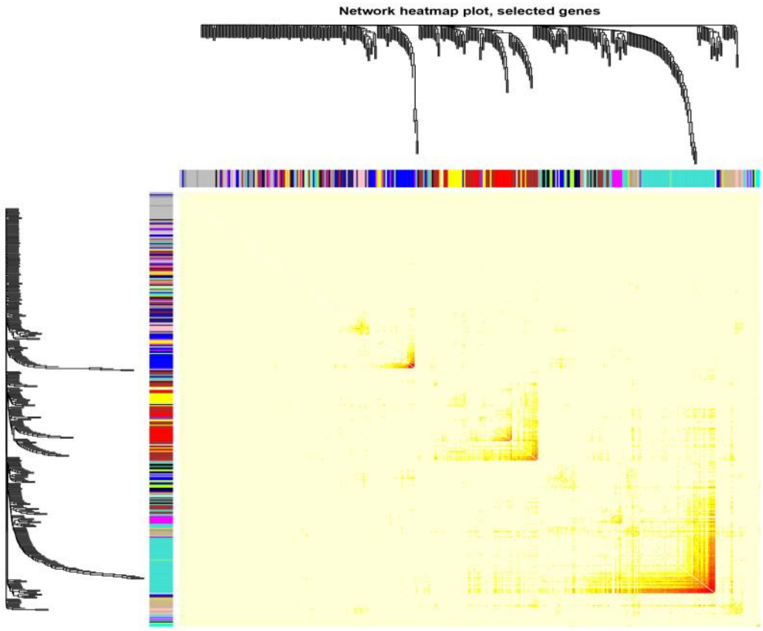

Supplementary Figure 22 | Visualization of the gene network using a heatmap plot. Heatmap depicts the topological overlap matrix (TOM) among all genes in the analysis. Light color represents low overlap and progressively darker red color represents higher overlap. Blocks of darker colors along the diagonal are the constructed modules. Gene dendrogram and module assignment are shown along the left side and the top.

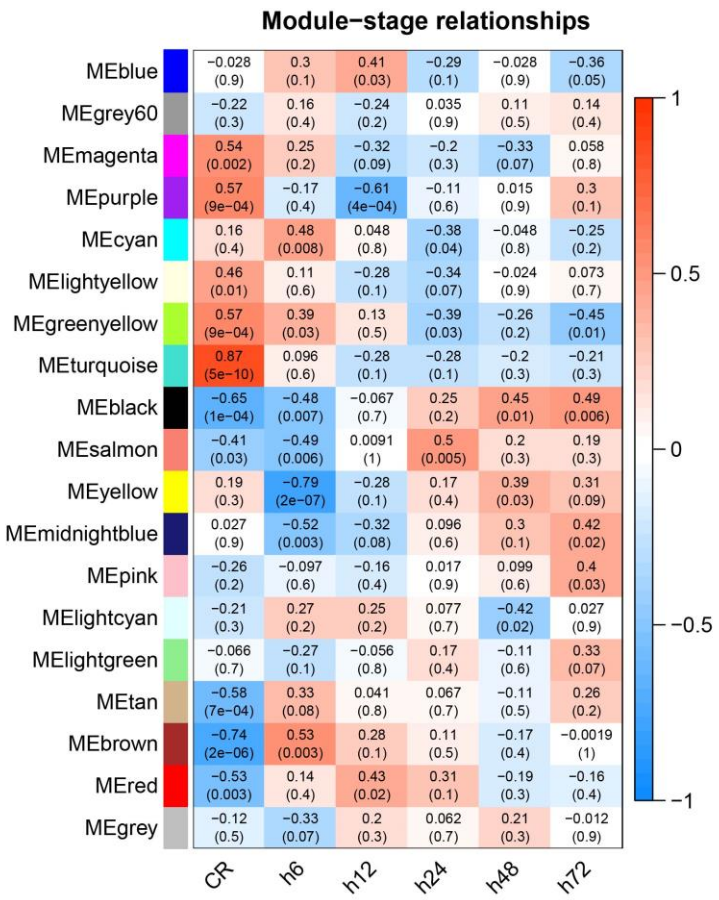

Supplementary Figure 23 | Module-regeneration-stage associations. Each row corresponds to a module eigengene and columns to a trait. Each cell contains the corresponding correlation and *P*-value. Table is color-coded by correlation according to the color legend.

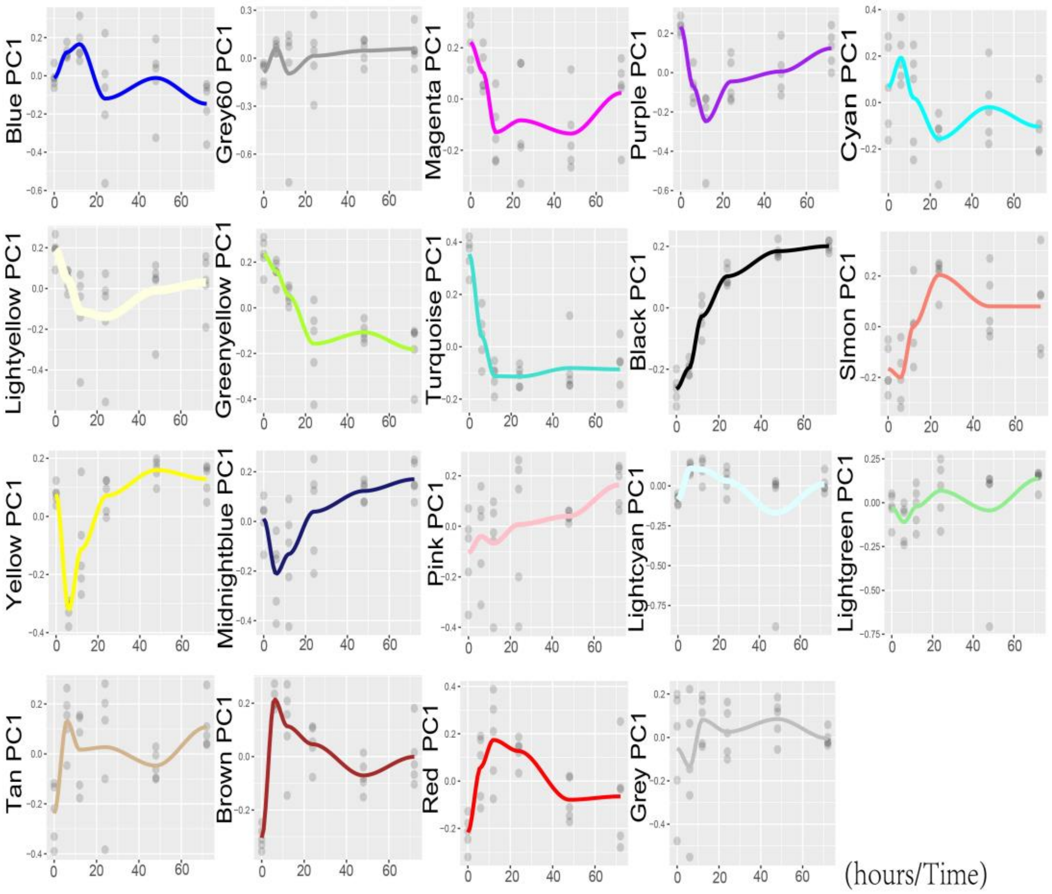

Supplementary Figure 24 | Characterization of the module trajectory throughout regeneration processes. The fit line represents locally weighted scatterplot smoothing. X-axis represents regenerative time points (hours). Y-axis represents module PC1 (Module-eigengene).

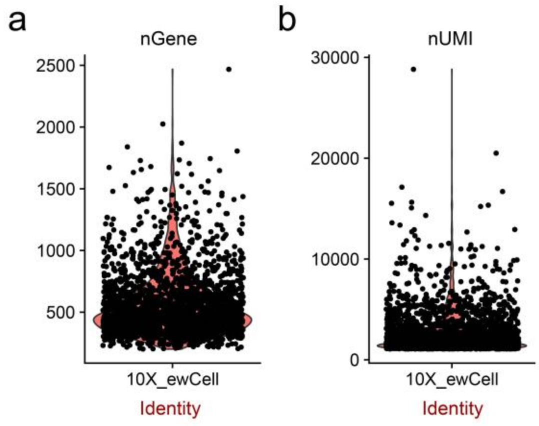

Supplementary Figure 25 | Number of genes and UMIs. The two plots for genes and nUMI in the earthworm single cell transcriptome**.** **a**, Number of genes. **b**, Number of UMIs.

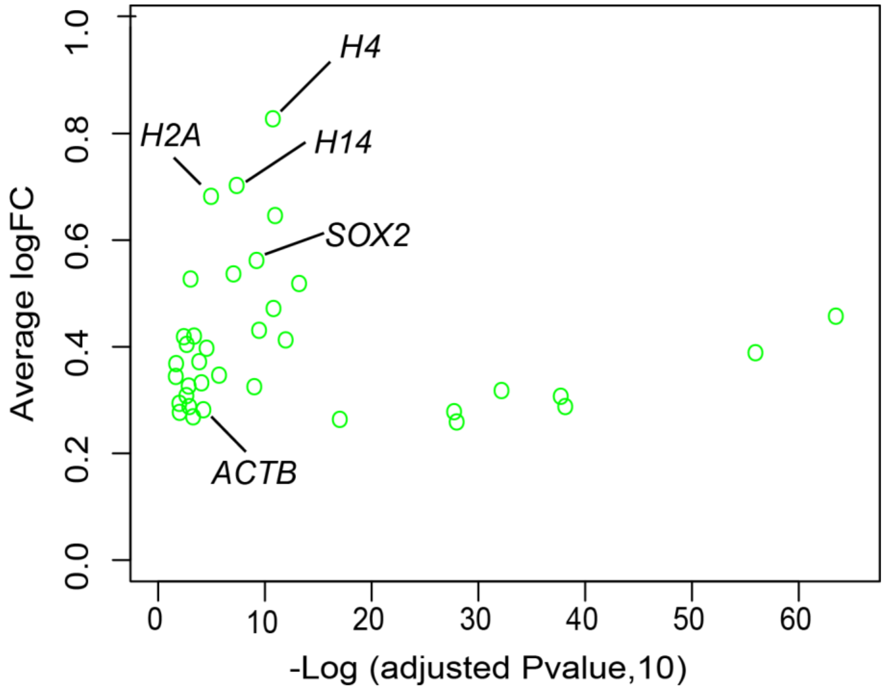

Supplementary Figure 26 | Expressions of gene markers in pluripotent stem cell cluster0. Scatter plot of identified markers for cluster0, including well-known pluripotent marker genes. X-axis represented normalized *P* value. Y-axis represented fold changes of gene expressions.

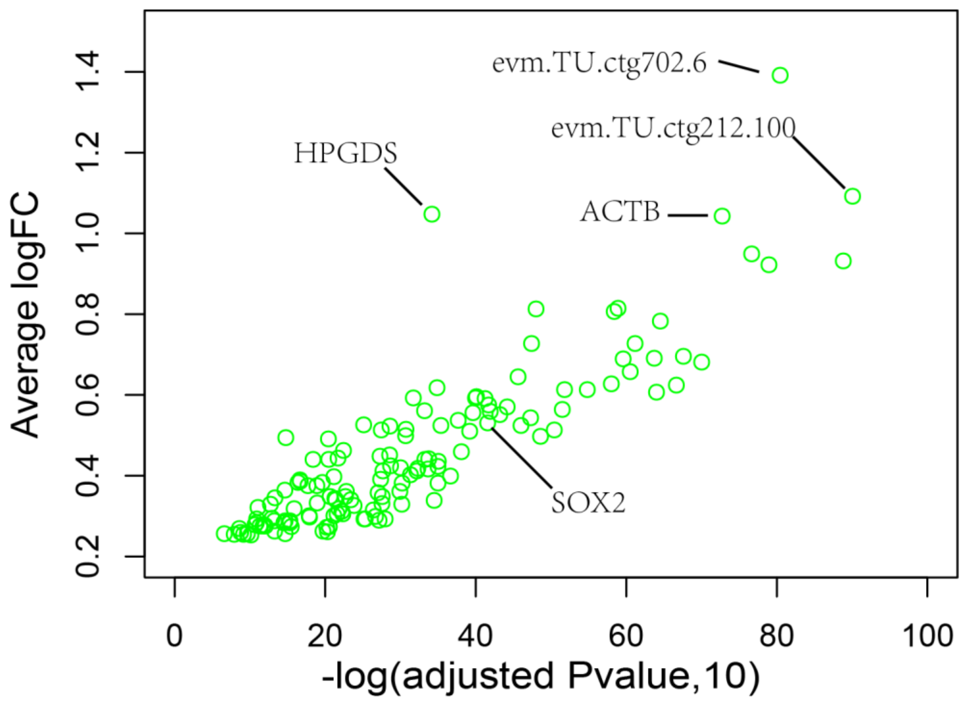

Supplementary Figure 27 | Expressions of gene markers in pluripotent stem cell cluster1. Scatter plot of identified markers for cluster1, including well-known pluripotent marker genes. X-axis represented normalized *P* value. Y-axis represented fold changes of gene expressions.

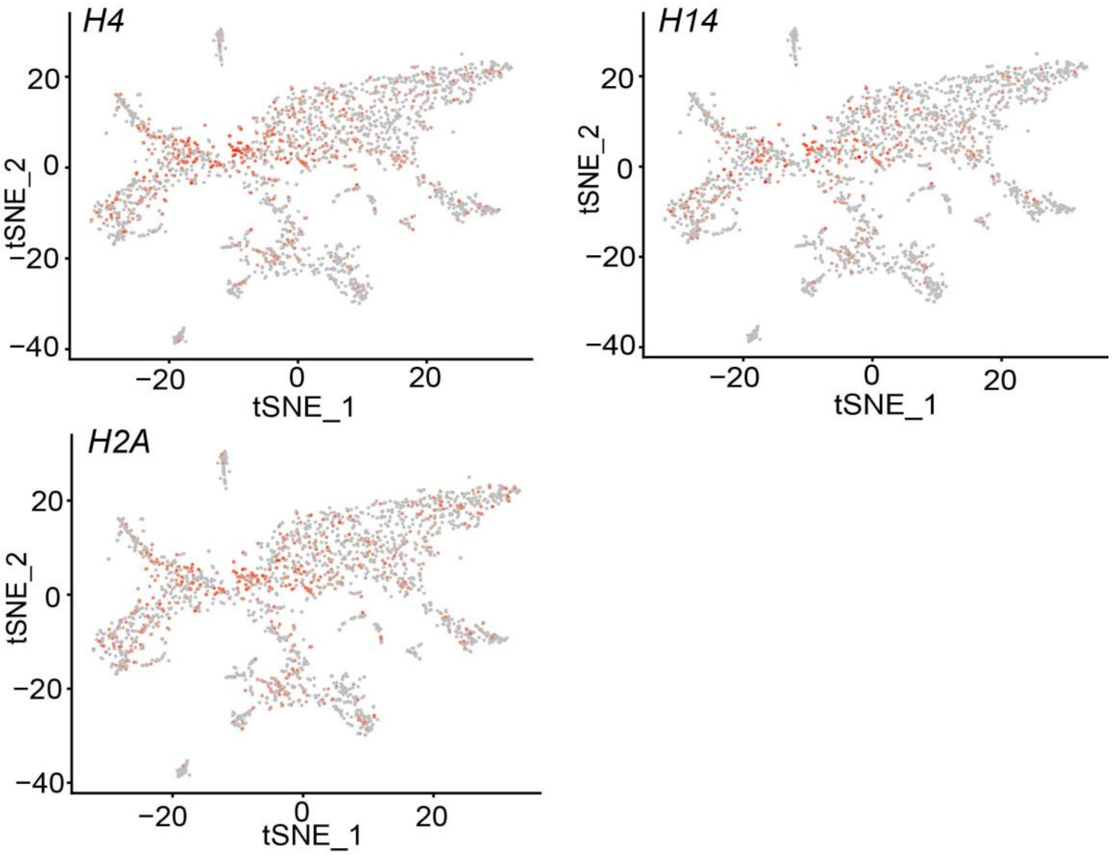

Supplementary Figure 28 | Expression of histone genes using the t-SNE plot. Cells expressing *H4*, *H14* and *H2A* were colored by red in different plots, respectively.

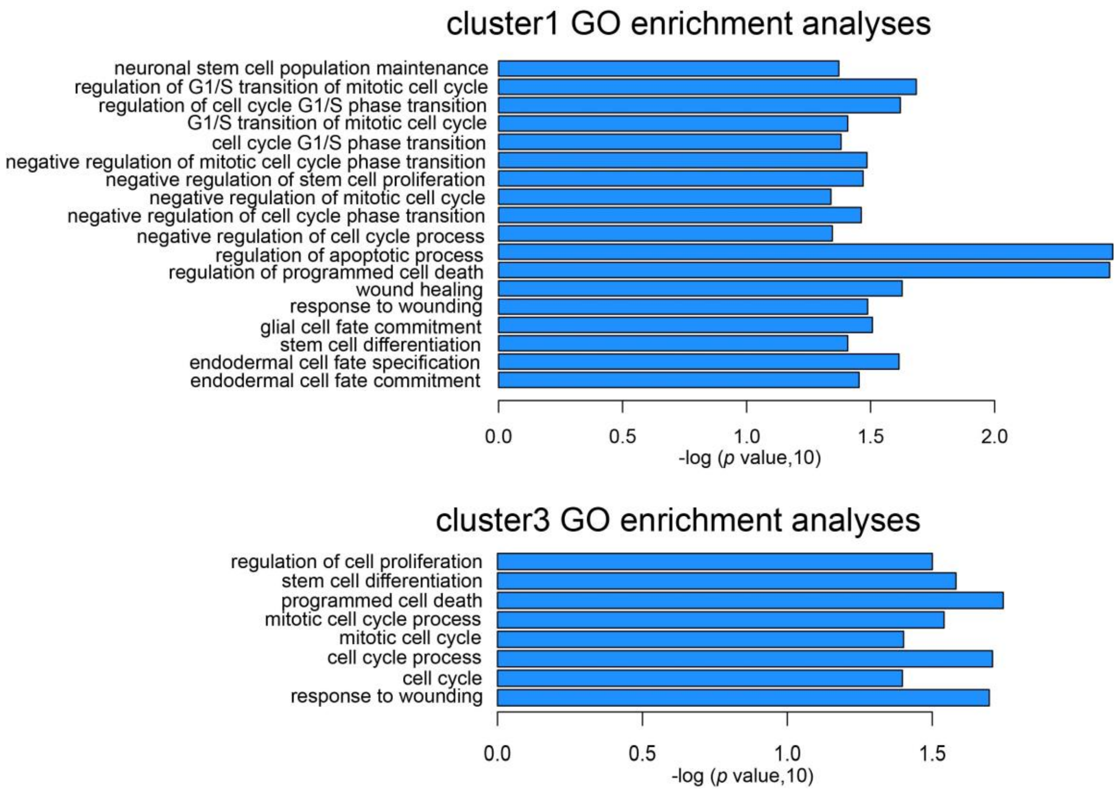

Supplementary Figure 29 | GO biological process enrichment analyses of highly expressed marker genes in clusters 1 and 3. GO enrichment analyses were performed by using g:Profiler software (https://biit.cs.ut.ee/). All known genes of statistical domain size using Homo sapiens were regarded as background and *p* values (Significance threshold) were adjusted by using Benjamini-Hochberg FDR.

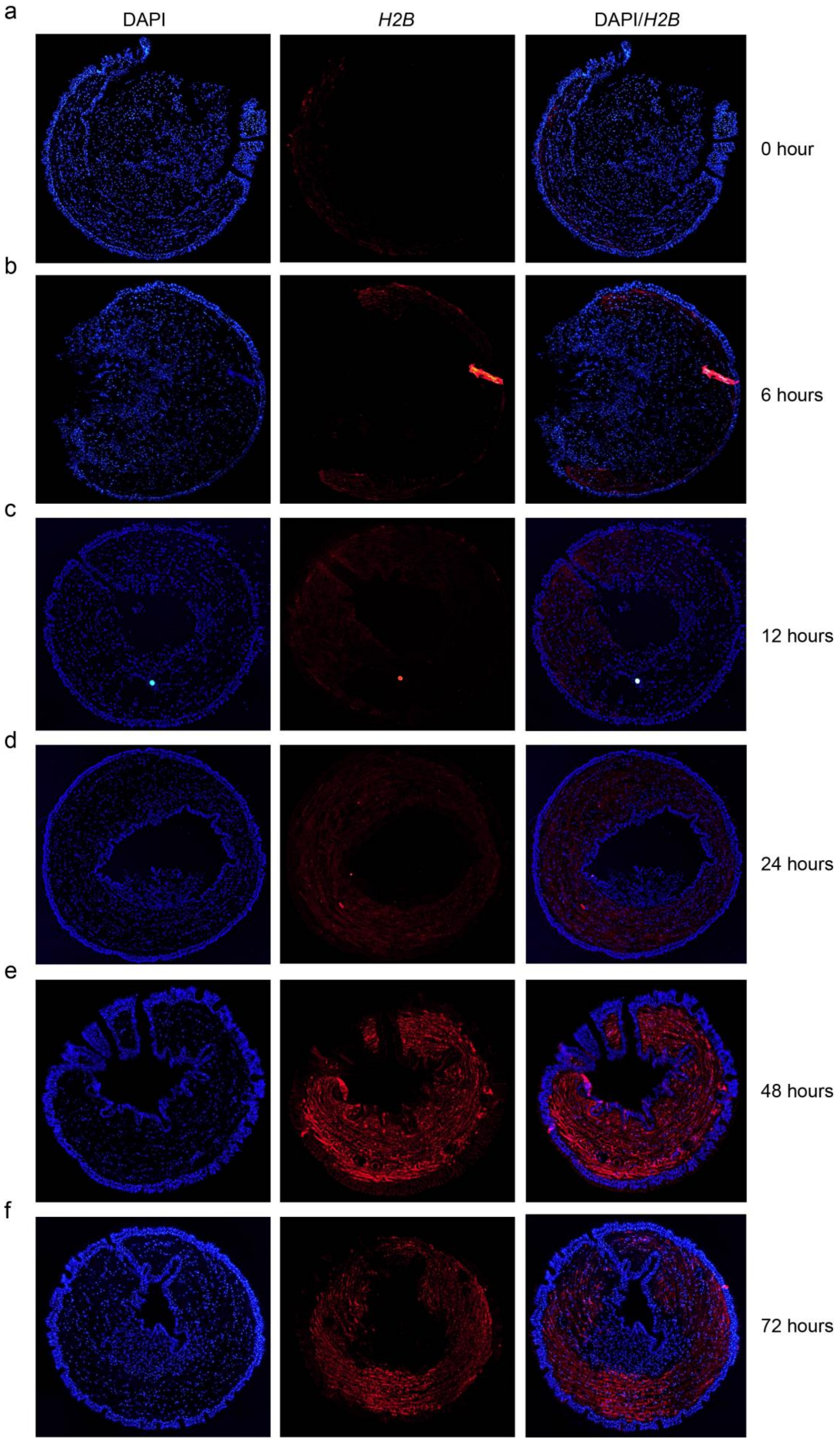
a
DAPI
H2B
DAPI/H2B
0 hour
b
6 hours
c
12 hours
d
24 hours
e
48 hours
f
72 hours

Supplementary Figure 30 | In situ hybridization of *H2B* in cross sections at 6 time points post-amputation for the earthworm. The slice size was 10μm. The 6 time points post-amputation included 0, 6, 12, 24, 48 and 72 hours. The red fluorescence represented positive signals and DAPI (blue fluorescence) was used to stain cell nucleus. **a**, 0 hour. **b**, 6 hours. **c**, 12 hours. **d**, 24 hours. **e**, 48 hours. **f**, 72 hours. Similar results in **a**, **b**, **c, d, e** and **f** could be reproduced under three independently biological experiments.

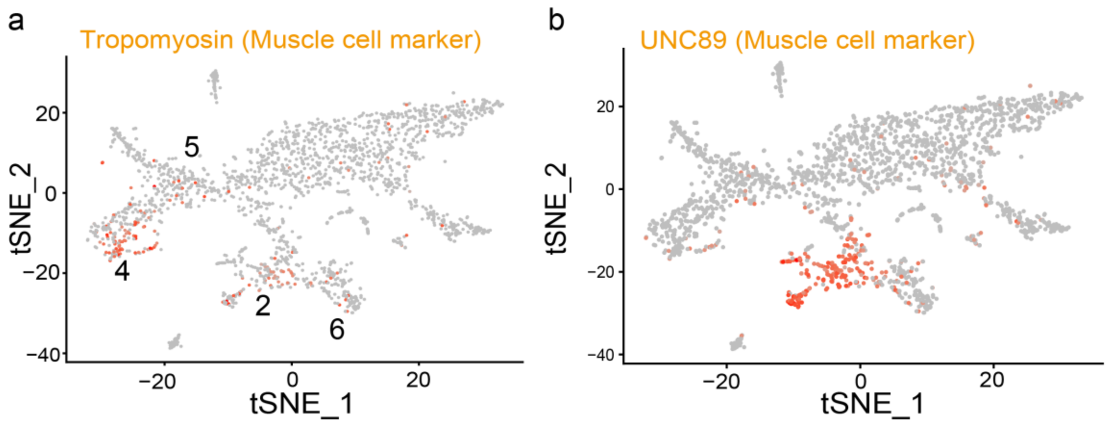

Supplementary Figure 31 | Characterizations of cluster 2 and 4 with published muscle cell maker gene with tSNE plot. a, *Tropomyosin.* **b**, *UNC89*.

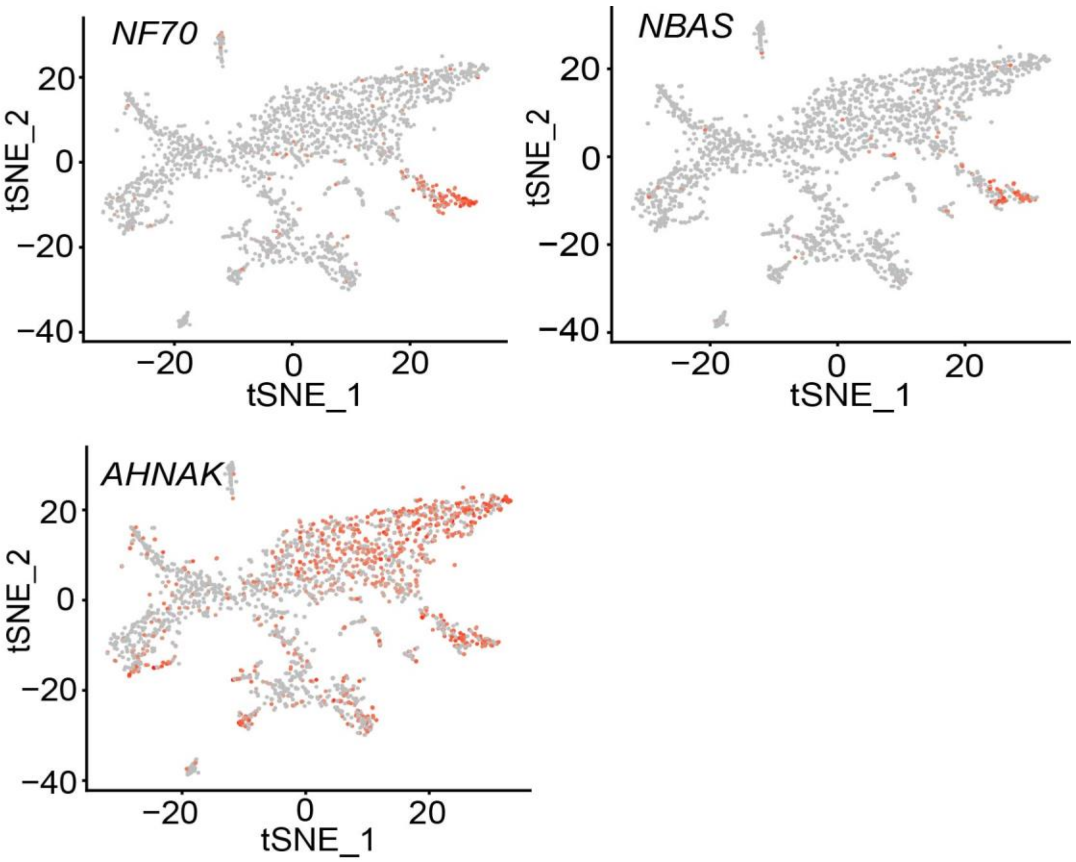

Supplementary Figure 32 | Characterization of cluster 7 with a neuronal cell maker gene with tSNE plot. The genes *NBAS*, *AHNAK* and *NF70*[1-3] playing roles in neural cells are neuronal cell markers.

	Wtdbg	FALCON	Canu
Genome size (Gb)	1.3	1.6	2.1
Longest contig (Mb)	6.0	3.3	5.7
Contig N50 (Kb)	740.8	225.1	255.2
Contig N90 (Kb)	65.1	62.7	50.3

Supplementary Table 1 | Comparison of earthworm genome assembly using Wtdbg, FALCON and Canu.

**Species**	**Genome size**	**Contig N50**	**Gene Number**	**Published Year**
Ciona intestinalis	160 Mb	20 Kb	16,000	2002
Strongylocentrotus purpuratus	814 Mb	18.5 Kb	23,300	2006
Nematostella vectensis	470 Mb	19.8 Kb	18,000	2007
Hydra magnipapillata	1.05 Gb	12.8 Kb	20,000	2010
Ectocarpus siliculosus	214 Mb	32 Kb	16,256	2010
Acropora digitifera	420 Mb	10 Kb	23,688	2011
Capitella teleta	324 Mb	21.9 Kb	32,389	2012
Crassostrea gigas	559 Mb	19.4 Kb	28,000	2012
Lottia gigantea	348 Mb	96 Kb	23,800	2012
Mnemiopsis leidyi	150 Mb	11.9 Kb	16,548	2013
Hippocampus comes	695 Mb	34.7 Kb	23,458	2016
Paralichthys olivaceus	546 Mb	30.5 Kb	21,787	2016
Patinopecten yessoensis	988 Mb	38 Kb	26,415	2017
Acanthaster planci	383 Mb	54.9 Kb	20,328	2017
Bathymodiolus platifrons	1.64 Gb	13.2 Kb	33,584	2017
Apostichopus japonicus	805 Mb	190.3Kb	30,350	2017
Macrostomum lighano	764Mb	215.3 Kb	49,018	2017
Eisenia andrei	**1.3 Gb**	**741 Kb**	**31,817**	**Present study**

Supplementary Table 2 | Assembly statistics of published invertebrate genomes.

Samples	Number of mapped assemblies	Number of assemblies	Ratio
12hr1	209,432	220,226	95.1%
24hr2	198,133	212,579	93.2%
48hr5	211,491	223,102	94.8%
6hr3	215,030	227,820	94.4%
72hr4	200,312	209,859	95.5%
CR3	225,239	233,332	96.5%

Supplementary Table 3 | Summary of six randomly selected earthworm transcriptomes assembled by Trinity.

	Complete	Fragmented	Missing
Number	901	13	64
Ratio	92.1%	1.3%	6.6%

Supplementary Table 4 | BUSCO analysis of the draft assembly.

Approach		Gene number	Total CDS length (Mb)	Average CDS length (bp)	Average exons per gene	Average exon length (bp)	Average intron length (bp)
De novo	Augustus	53,171	75.46	1,419	5.56	255	2,261
	SNAP	53,242	64.26	1,206	5.41	223	3,298
**Homology**	*C. teleta*	33,558	24.77	738	4.18	176	1,453
	H. robusta	24,970	18.77	751	4.03	186	1,460
**Transcriptome**	Cufflinks	46,765	145.12	3,103	5.52	562	2,409
**EVM**		31,817	56.37	1,771	8.03	221	2,333

Supplementary Table 5 | Statistics of predicted protein-coding genes in the earthworm genome.

Category	Number	Ratio
GO	19,006	59.7%
InterPro	24,498	77.0%
KEGG	24,467	76.9%
SwissProt	21,717	68.3%
Trembl	26,535	83.4%
All	27,728	87.1%

Supplementary Table 6 | Functional annotation of protein-coding genes in diverse databases.

Category	Number	Length(bp)
tRNA	1,292	102,608
rRNA	34	21,880
miRNA	1,244	154,131
snRNA	234	37,788
All	2,804	316,407

Supplementary Table 7 | Summary of predicted non-coding RNA genes in the earthworm genome.

	Repbase TEs		RepeatModeler TEs		Combined TEs	
	**Length**(bp)	**Ratio**(%)	**Length**(bp)	**Ratio**(%)	**Length**(bp)	**Ratio**(%)
DNA	2,175,695	0.17	187,691,577	14.27	186,269,725	14.16
LINE	13,039,549	0.99	163,564,336	12.43	163,423,637	12.42
LTR	332,512	0.03	30,978,179	2.35	30,145,435	2.29
SINE	1,367,910	0.10	7,637,895	0.58	7,718,111	0.59
Other	66,712,812	5.07	69,920,579	5.31	74,854,175	5.69
Unknown	332,064	0.03	284,504,576	21.62	283,875,506	21.58
Total	83,960,542	6.38	744,297,142	56.57	746,286,589	56.72

Supplementary Table 8 | Analysis of transposable elements in the earthworm genome.

FDR	Gene Number	GO ID	GO Taxonomy	GO Name
4.30E-02	3	GO:0016266	BP	O-glycan processing
2.80E-02	3	GO:0071427	BP	mRNA-containing ribonucleoprotein complex export from nucleus
2.80E-02	3	GO:0006406	BP	mRNA export from nucleus
2.58E-02	12	GO:0070647	BP	protein modification by small protein conjugation or removal
1.69E-02	8	GO:0002376	BP	immune process
1.51E-02	8	GO:0006955	BP	immune response
2.80E-02	6	GO:0009605	BP	response to external stimulus
1.81E-02	7	GO:0051704	BP	multi-organism process
1.06E-02	104	GO:0051179	BP	localization
1.30E-02	102	GO:0051234	BP	establishment of localization
1.60E-02	101	GO:0006810	BP	transport
6.67E-06	63	GO:0006811	BP	ion transport
2.70E-05	38	GO:0006812	BP	cation transport
1.02E-06	32	GO:0015672	BP	monovalent inorganic cation transport
1.66E-05	33	GO:0030001	BP	metal ion transport
8.86E-09	25	GO:0006814	BP	sodium ion transport
4.66E-02	26	GO:0034220	BP	ion transmembrane transport
3.60E-02	2	GO:0000186	BP	activation of MAPKK activity
1.92E-02	6	GO:0071526	BP	semaphorin-plexin signaling pathway
3.60E-02	2	GO:0032456	BP	endocytic recycling
2.69E-02	15	GO:0032501	BP	multicellular organismal process
4.79E-02	10	GO:0007275	BP	multicellular organism development
4.30E-02	4	GO:0009617	BP	response to bacterium
4.30E-02	4	GO:0042742	BP	defense response to bacterium

Supplementary Table 9 | GO enrichment analysis of unique gene families in earthworm.

Gene ID	Primer Name	Primer sequence (5’ to 3’)
evm.TU.ctg3.9	ctg3.9_F	GCAGCCGTTGTGTTTCCAAT
	ctg3.9_R	GACATCTTGCGGTGCAGTTG
evm.TU.ctg982.9	ctg982.9_F	GAAGTCCACCTCAGCAGACC
	ctg982.9_R	TGGTTGAGGCTCGATGGTTC
evm.TU.ctg408.9	ctg408.9_F	CCCATCAAGTGGTTGGCTCT
	ctg408.9_R	AGGGCTTGTGGAATCTGTCG
evm.TU.ctg646.3	ctg646.3_F	GCGTCAAAATCCTCGGCATC
	ctg646.3_R	GCAGTGAACAATCCCCCGTA
evm.TU.ctg683.28	ctg683.28_F	GATGTCGAATGTGTCGCGTG
	ctg683.28_R	GCGGGATCAAGGTAGACGAG
evm.TU.ctg91.63	ctg91.63_F	CAAGCATGTGCCGAATCCAG
	ctg91.63_R	CTGTGCACTGTCTGGGACAT
evm.TU.ctg94.37	ctg94.37_F	ATCGTTACTCCGACCGATGC
	ctg94.37_R	GAGTTGGTCGCCTCTGTCAA
evm.TU.ctg408.8	ctg408.8_F	ATACGATCCGCGAGGTCAAC
	ctg408.8_R	TGCAATTCCTTGAGCCCGAT
β-actin (Reference)	β-actin_F	ACGAAGGTTATGCTCTGCCACAC
	β-actin_R	CTCTTTCGGCTGTCGTCGTGAAG

Supplementary Table 10 | Primers of qPCR for EGFR copies.

Gene ID	Homologues	Symbol	moduleColor	MM.brown	p.MM.brown
evm.TU.ctg192.7	CapteG176110	MYPH	brown	0.960478678	4.36E-17
evm.TU.ctg539.23	CapteG170504	FOS	brown	0.958639506	8.15E-17
evm.TU.ctg299.5	CapteG178665	DYRK2	brown	0.9550762	2.54E-16
evm.TU.ctg549.8	CapteG176380	GRB10	brown	0.953598537	3.95E-16
evm.TU.ctg103.23	CapteG170392	FHL2	brown	0.949444432	1.28E-15
evm.TU.ctg114.32	CapteG177478	PEBB	brown	0.947492433	2.15E-15
evm.TU.ctg19.4	CapteG228569	MMP14	brown	0.945766874	3.34E-15
evm.TU.ctg1175.3	CapteG197066	RHG20	brown	0.94349469	5.85E-15
evm.TU.ctg837.1	CapteG186269		brown	0.941505506	9.38E-15
evm.TU.ctg130.42	CapteG97875	SL9A2	brown	0.938900249	1.70E-14
evm.TU.ctg683.28	CapteG226426	EGFR	brown	0.937991074	2.08E-14
evm.TU.ctg142.55	CapteG156731	TPM1	brown	0.937583191	2.27E-14
evm.TU.ctg423.23	CapteG171705	DCMC	brown	0.936693684	2.75E-14
evm.TU.ctg127.15	CapteG215426	MFS4B	brown	0.93606517	3.15E-14
evm.TU.ctg530.3	CapteG199858	HUNB	brown	0.934630273	4.26E-14
evm.TU.ctg238.49	CapteG172802	MEIOC	brown	0.9321239	7.10E-14
evm.TU.ctg81.18	CapteG186269	DDT4L	brown	0.932031485	7.23E-14
evm.TU.ctg72.89	CapteG50343	TISB	brown	0.931872398	7.47E-14
evm.TU.ctg299.19	CapteG158933	KGP1	brown	0.930986019	8.90E-14
evm.TU.ctg983.6	CapteG103587	SIK3	brown	0.929800071	1.12E-13
evm.TU.ctg332.4	CapteG77182	SOCS3	brown	0.929140722	1.27E-13
evm.TU.ctg588.6	CapteG171008	MOT5	brown	0.928653397	1.40E-13
evm.TU.ctg296.54	CapteG226569	KCNJ6	brown	0.927066609	1.88E-13
evm.TU.ctg272.6	CapteG162250	S13A2	brown	0.925976198	2.30E-13
evm.TU.ctg123.16	CapteG227995	GALE	brown	0.92434761	3.09E-13
evm.TU.ctg698.2	CapteG194504	T151B	brown	0.924292218	3.12E-13
evm.TU.ctg302.1	CapteG195496	CRERF	brown	0.924020985	3.27E-13
evm.TU.ctg210.11	CapteG198404		brown	0.923644255	3.50E-13
evm.TU.ctg530.2	CapteG199858	HUNB	brown	0.922179135	4.52E-13
evm.TU.ctg351.2	CapteG227359	REST	brown	0.921760129	4.87E-13
evm.TU.ctg1275.5	CapteG222792		brown	0.92130248	5.26E-13
evm.TU.ctg359.5	CapteG169335	BRD2	brown	0.920181555	6.37E-13
evm.TU.ctg32.20	CapteG220321	HVCN1	brown	0.918829033	7.99E-13
evm.TU.ctg30.33	CapteG163268	GDS1A	brown	0.918618546	8.28E-13
evm.TU.ctg29.34	CapteG213150	FMAR	brown	0.918072125	9.06E-13
evm.TU.ctg100.23	CapteG50343	TISB	brown	0.917398793	1.01E-12
evm.TU.ctg364.3	CapteG127570	PDC6I	brown	0.917000979	1.08E-12
evm.TU.ctg432.9	CapteG206255	HMHA1	brown	0.916193032	1.23E-12
evm.TU.ctg499.11	CapteG226667	ELL2	brown	0.915855333	1.30E-12
evm.TU.ctg3186.1	CapteG228725	ATS18	brown	0.913955352	1.75E-12
evm.TU.ctg266.72	CapteG77182	SOCS3	brown	0.913702247	1.82E-12
evm.TU.ctg192.6	CapteG227370	TNAP3	brown	0.91354691	1.87E-12

evm.TU.ctg137.13	CapteG129833	SHAN3	brown	0.912650687	2.15E-12
evm.TU.ctg71.3			brown	0.910865027	2.82E-12
evm.TU.ctg745.4	CapteG105015	PPM1D	brown	0.909878695	3.27E-12
evm.TU.ctg325.14		ACK1	brown	0.90962553	3.39E-12
evm.TU.ctg348.2	CapteG174485	S23A1	brown	0.908604045	3.95E-12
evm.TU.ctg72.93	CapteG220507	CO6A4	brown	0.908395067	4.07E-12
evm.TU.ctg975.7	CapteG174539	UNC9	brown	0.908282468	4.14E-12
evm.TU.ctg310.25	CapteG158679	ACT2	brown	0.90712193	4.90E-12

Supplementary Table 11 | Genes in the brown module with a higher intramodule membership during regeneration. Here, top50 genes with Intramodule membership were showed.

FDR	Number	term ID	Class	GO Name
4.99E-02	4	GO:0042558	BP	pteridine-containing compound metabolic process
2.49E-02	10	GO:0006813	BP	potassium ion transport
3.64E-02	3	GO:0046653	BP	tetrahydrofolate metabolic process
4.03E-02	50	GO:0006796	BP	phosphate-containing compound metabolic process
8.70E-04	37	GO:0016310	BP	phosphorylation
1.79E-04	35	GO:0006468	BP	protein phosphorylation
4.25E-02	15	GO:0007166	BP	cell surface receptor signaling pathway
4.03E-02	5	GO:0051336	BP	regulation of hydrolase activity
3.10E-02	27	GO:0071944	CC	cell periphery
3.66E-02	26	GO:0005886	CC	plasma membrane
2.91E-03	18	GO:0044459	CC	plasma membrane part
3.10E-03	12	GO:0031226	CC	intrinsic component of plasma membrane
3.10E-03	12	GO:0005887	CC	integral component of plasma membrane
4.86E-02	6	GO:0016209	MF	antioxidant activity
3.64E-02	230	GO:0003824	MF	catalytic activity
1.33E-03	75	GO:0140096	MF	catalytic activity, acting on a protein
4.00E-02	91	GO:0016740	MF	transferase activity
7.56E-05	49	GO:0016772	MF	transferase activity, transferring phosphorus-containing groups
3.88E-06	44	GO:0016773	MF	phosphotransferase activity, alcohol group as acceptor
1.38E-05	45	GO:0016301	MF	kinase activity
3.64E-02	4	GO:0003951	MF	NAD+ kinase activity
7.56E-05	36	GO:0004672	MF	protein kinase activity
2.35E-04	18	GO:0004674	MF	protein serine/threonine kinase activity
1.99E-02	51	GO:0016491	MF	oxidoreductase activity
4.99E-02	5	GO:0016684	MF	oxidoreductase activity, acting on peroxide as acceptor
2.01E-03	371	GO:0005488	MF	binding
3.88E-06	179	GO:0043167	MF	ion binding
3.62E-05	98	GO:0043168	MF	anion binding
2.26E-03	82	GO:0097367	MF	carbohydrate derivative binding
3.50E-05	99	GO:0036094	MF	small molecule binding
2.35E-04	89	GO:1901265	MF	nucleoside phosphate binding
2.48E-03	75	GO:0035639	MF	purine ribonucleoside triphosphate binding
2.35E-04	89	GO:0000166	MF	nucleotide binding
2.26E-03	76	GO:0032553	MF	ribonucleotide binding
2.53E-03	75	GO:0017076	MF	purine nucleotide binding
1.46E-02	60	GO:0030554	MF	adenyl nucleotide binding
2.48E-03	75	GO:0032555	MF	purine ribonucleotide binding
1.46E-02	60	GO:0032559	MF	adenyl ribonucleotide binding
1.46E-02	60	GO:0005524	MF	ATP binding
4.03E-02	9	GO:0015079	MF	potassium ion transmembrane transporter activity
4.86E-02	7	GO:0022843	MF	voltage-gated cation channel activity

Supplementary Table 12 | GO enrichment analysis of the black module.

Gene ID	Homologue	Symbol	moduleColor	MM.black	p.MM.black
evm.TU.ctg1539.1	CapteG71935	AGRIN	black	0.927632028	1.69E-13
evm.TU.ctg1025.11	CapteG196162		black	0.92156417	5.03E-13
evm.TU.ctg413.26	CapteG210699		black	0.916257732	1.22E-12
evm.TU.ctg262.27	CapteG170895	MOT14	black	0.90865928	3.91E-12
evm.TU.ctg81.61	CapteG126517	SPHK1	black	0.903903928	7.73E-12
evm.TU.ctg1215.3	CapteG170895	MOT9	black	0.902516591	9.36E-12
evm.TU.ctg343.17	CapteG219042	MLXPL	black	0.899589522	1.39E-11
evm.TU.ctg150.21	CapteG71935	AGRIN	black	0.89904043	1.50E-11
evm.TU.ctg433.28	CapteG160399		black	0.893244394	3.15E-11
evm.TU.ctg424.4	CapteG222921	APLP	black	0.879484608	1.57E-10
evm.TU.ctg1974.3	CapteG228443	FAT3	black	0.878402065	1.77E-10
evm.TU.ctg685.15		LECT1	black	0.877133362	2.03E-10
evm.TU.ctg24.84	CapteG164804	PDIA3	black	0.875340018	2.46E-10
evm.TU.ctg685.11		LECT1	black	0.873669394	2.93E-10
evm.TU.ctg19.34	CapteG210005	S5A3A	black	0.867965255	5.25E-10
evm.TU.ctg322.1	CapteG158885	SING	black	0.867230292	5.65E-10
evm.TU.ctg409.3	CapteG154368	ATG2B	black	0.86329124	8.29E-10
evm.TU.ctg130.38	CapteG147868	KPRA	black	0.856932187	1.50E-09
evm.TU.ctg776.2	CapteG188525	RFIP2	black	0.851750911	2.39E-09
evm.TU.ctg164.59			black	0.848179613	3.26E-09
evm.TU.ctg3762.1		LECT1	black	0.846851663	3.65E-09
evm.TU.ctg238.36	CapteG149382		black	0.840645498	6.12E-09
evm.TU.ctg27.46	CapteG108481	PTK7	black	0.83739079	7.95E-09
evm.TU.ctg546.9	CapteG164703	MYG1	black	0.836889809	8.27E-09
evm.TU.ctg396.1	CapteG216514	EKI1	black	0.835919894	8.93E-09
evm.TU.ctg306.29	CapteG220701	MOT12	black	0.834612032	9.90E-09
evm.TU.ctg1025.14	CapteG196162		black	0.833229492	1.10E-08
evm.TU.ctg485.48	CapteG221849	MRF	black	0.831244899	1.28E-08
evm.TU.ctg1123.2	CapteG127905	S13A3	black	0.83092322	1.32E-08
evm.TU.ctg74.69	CapteG159655	IQGA1	black	0.826059715	1.90E-08
evm.TU.ctg406.56	CapteG173272	CCD22	black	0.820860291	2.77E-08
evm.TU.ctg2.43	CapteG98040	CANA	black	0.81999187	2.94E-08
evm.TU.ctg13.44	CapteG89730	LIN41	black	0.817579845	3.49E-08
evm.TU.ctg299.7	CapteG171427	GLCTK	black	0.816461319	3.78E-08
evm.TU.ctg156.36	CapteG177803	FURIN	black	0.811949762	5.15E-08
evm.TU.ctg489.3	CapteG198861	FBCDB	black	0.80947754	6.08E-08
evm.TU.ctg101.26	CapteG140091	THOC1	black	0.808996431	6.28E-08
evm.TU.ctg389.2	CapteG228655	WDFY2	black	0.807329321	7.01E-08
evm.TU.ctg426.23	CapteG179070	HIP1	black	0.804359708	8.52E-08
evm.TU.ctg305.46	CapteG100629	TBC15	black	0.803706772	8.89E-08
evm.TU.ctg111.27	CapteG214729	STX7	black	0.801541139	1.02E-07
evm.TU.ctg1377.4	CapteG222999		black	0.799009101	1.20E-07

evm.TU.ctg356.10	CapteG228725	ATS6	black	0.79737091	1.33E-07
evm.TU.ctg81.72			black	0.796342163	1.42E-07
evm.TU.ctg720.5	CapteG51996	MYO3B	black	0.795927064	1.45E-07
evm.TU.ctg1322.3	CapteG213869	UBP47	black	0.793332004	1.71E-07
evm.TU.ctg10.8	CapteG227168	AAPK2	black	0.793247943	1.71E-07
evm.TU.ctg423.26	CapteG120325	NRG	black	0.790854189	1.98E-07
evm.TU.ctg575.9	CapteG186677	TBCD9	black	0.789513631	2.15E-07

Supplementary Table 13 | Genes in the black module with a higher intramodule membership during regeneration. Here, top 50 genes with positive intramodule membership were showed.

	Numerical value
Estimated Number of Cells	2,080
Fraction Reads in Cells	57.10%
Mean Reads per Cell	200,874
Median Genes per Cell	493
Total Genes Detected	21,878
Median UMI Counts per Cell	1,904

Supplementary Table 14 | Data quality statistics for single-cell RNA-sequencing.

Supplementary References

1. McMillan, E.L., Kamps, A.L., Lake, S.S., Svendsen, C.N. & Bhattacharyya, A. Gene expression changes in the MAPK pathway in both Fragile X and Down syndrome human neural progenitor cells. *Am J Stem Cells* **1**, 154-162 (2012).
2. Chan, S.F., Huang, X., McKercher, S.R., Zaidi, R., Okamoto, S.I., Nakanishi, N. *et al.* Transcriptional profiling of MEF2-regulated genes in human neural progenitor cells derived from embryonic stem cells. *Genom Data* **3**, 24-27 (2015).
3. Ning, H., Lin, G., Lue, T.F. & Lin, C.S. Neuron-like differentiation of adipose tissue-derived stromal cells and vascular smooth muscle cells. *Differentiation* **74**, 510-518 (2006).
