# Supplementary material for: Genome and single-cell RNA-sequencing of the earthworm Eisenia andrei identifies cellular mechanisms underlying regeneration
Source: Nat Commun. 2020 May 27;11:2656. doi: 10.1038/s41467-020-16454-8 (PMC7253469; doi:10.1038/s41467-020-16454-8)
Supplement: Supplementary file 9 — Description of Additional Supplementary Files [file 41467_2020_16454_MOESM9_ESM.pdf]

Title: Supplementary Data 1.
Description: Overlapping of enriched functional categories for differentially expressed up-regulated genes across regenerative stages.

Title: Supplementary Data 2.
Description: Statistics of transposable elements in five invertebrate genomes.

Title: Supplementary Data 3.
Description: GO enrichment analysis of 2776 significantly expanded gene families in earthworm.

Title: Supplementary Data 4.
Description: GO enrichment analysis of the brown module.

Title: Supplementary Data 5.
Description: GO enrichment analysis of the red module.

Title: Supplementary Data 6.
Description: GO enrichment analysis of the blue module.
