# Supplementary material for: Genome and single-cell RNA-sequencing of the earthworm Eisenia andrei identifies cellular mechanisms underlying regeneration
Source: Nat Commun. 2020 May 27;11:2656. doi: 10.1038/s41467-020-16454-8 (PMC7253469; doi:10.1038/s41467-020-16454-8)
Supplement: Supplementary file 10 — Reporting Summary [file 41467_2020_16454_MOESM10_ESM.pdf]

nature research

Corresponding author(s): Dong-Dong Wu, Jue Ruan, and Guo-Jie Zhang

Last updated by author(s): Mar 9, 2020

Reporting Summary

Nature Research wishes to improve the reproducibility of the work that we publish. This form provides structure for consistency and transparency in reporting. For further information on Nature Research policies, seeAuthors & Referees and theEditorial Policy Checklist .

Statistics

For all statistical analyses, confirm that the following items are present in the figure legend, table legend, main text, or Methods section.

n/a	Confirmed	
☐	☒	The exact sample size (*n*) for each experimental group/condition, given as a discrete number and unit of measurement
☐	☒	A statement on whether measurements were taken from distinct samples or whether the same sample was measured repeatedly
☐	☒	The statistical test(s) used AND whether they are one- or two-sided<br>*Only common tests should be described solely by name; describe more complex techniques in the Methods section.*
☒	☐	A description of all covariates tested
☐	☒	A description of any assumptions or corrections, such as tests of normality and adjustment for multiple comparisons
☐	☒	A full description of the statistical parameters including central tendency (e.g. means) or other basic estimates (e.g. regression coefficient) AND variation (e.g. standard deviation) or associated estimates of uncertainty (e.g. confidence intervals)
☒	☐	For null hypothesis testing, the test statistic (e.g. *F*, *t*, *r*) with confidence intervals, effect sizes, degrees of freedom and *P* value noted<br>*Give P values as exact values whenever suitable.*
☒	☐	For Bayesian analysis, information on the choice of priors and Markov chain Monte Carlo settings
☒	☐	For hierarchical and complex designs, identification of the appropriate level for tests and full reporting of outcomes
☒	☐	Estimates of effect sizes (e.g. Cohen's *d*, Pearson's *r*), indicating how they were calculated

Our web collection on statistics for biologists contains articles on many of the points above.

Software and code

Policy information about availability of computer code

Data collection	The raw genome sequencing data was collected by using PacBio RS and Illumina platforms. The raw RNA-Seq data at different stages was produced by Illumina Hiseq platform. And the raw single-cell RNA-Sequencing data was from 10X Genomics platform.
Data analysis	We involved in tools in data analysis: Wtdbg (v1.2.7), FALCON (v052016), Canu (v1.7), Arrow (v2.3.2), Pilon (v1.22), Trinity (v2.1.1), RepeatModeler (v1.0.8), RepeatMasker (v4.0.7), RepBase (v20150807), Augustus (v3.0.3), SNAP (v2006-07-28), PASA (r20140417), genBlastA (v1.0.138), GeneWise (v2.2.3), TopHat2 (v2.0.13), Cufflinks (v2.1.1), EVidenceModeler (EVM) (r2012-06-25), BLASTP (ncbi-blast-2.2.28+), InterProScan (v5.21-60.0), OrthoMCL (v2.0.9), CAFE (v2.2), MUSCLE (v3.8.31), RAxML (v8.2.9), PAML (v4.8a), DESeq2 (v1.26.0), R (v3.5.1), WGCNA (v1.67), VisANT (v5.0), 10X Genomics Cell Ranger (v2.1.1), Seurat (v2), Monocle (v1) and g:Profiler (rev1705). Note that TimeTree (http://www.timetree.org/) is a web-based database without a version information.

For manuscripts utilizing custom algorithms or software that are central to the research but not yet described in published literature, software must be made available to editors/reviewers. We strongly encourage code deposition in a community repository (e.g. GitHub). See the Nature Research guidelines for submitting code & software for further information.

Data

Policy information about availability of data

All manuscripts must include a data availability statement. This statement should provide the following information, where applicable:

- Accession codes, unique identifiers, or web links for publicly available datasets
- A list of figures that have associated raw data
- A description of any restrictions on data availability

The genome assembly and annotation data have been submitted to Genome Warehouse in National Genomics Data Center (http://bigd.big.ac.cn/gwh/) under accession code GWHACBE00000000. The genome sequencing data have been deposited at Sequence Read Archive (SRA) database in National Center for Biotechnology Information (NCBI) with accession code PRJNA541361. The transcriptome sequencing data have been deposited at NCBI SRA database under accession code PRJNA541362. The single-cell transcriptome sequencing data have been demonstrated in NCBI SRA database with accession code PRJNA541363.

Field-specific reporting

Please select the one below that is the best fit for your research. If you are not sure, read the appropriate sections before making your selection.

- [x] Life sciences
- [ ] Behavioural & social sciences
- [ ] Ecological, evolutionary & environmental sciences

For a reference copy of the document with all sections, see nature.com/documents/nr-reporting-summary-flat.pdf

Life sciences study design

All studies must disclose on these points even when the disclosure is negative.

Sample size	We selected one earthworm individual to finish de novo whole genome sequencing and assembly. For RNA-seq, we provided 5 biological replicates for each stage to perform further transcriptome analyses because of RNA-seq technology possessing a high sensitivity in detecting gene expression abundance. For many transcriptomics studies (RNA-seq), such as references (chicken in Wang et al. 2016; pistachio in Zeng et al. 2019; pigeon in Shao et al. 2020), generally, each experimental condition possesses >=3 biological replicates to detect differentially expressed genes. In this study, we provided more biological replicates (5 biological replicates) than listed references to detect gene expression. However, for single-cell transcriptome sequencing, we provided a pool sample library at the cross section after regenerative 72 h using multiple earthworm individuals with same conditions because we only concerned cell types and their proportions.
Data exclusions	No data were excluded from the analyses.
Replication	All experiments (e.g. HE Staining and cell proliferation) can be repeatedly validated by at least two independently biological experiments in this manuscript. For transcriptome sequencing analyses, 5 biological replicates were given for each stage. Further, our transcriptome analyses could be validated by using qPCR experiments, such as egfr.
Randomization	We randomly selected samples from hundreds of the earthworm individuals.
Blinding	Analyses and experiments are blinding in this study.

Reporting for specific materials, systems and methods

We require information from authors about some types of materials, experimental systems and methods used in many studies. Here, indicate whether each material, system or method listed is relevant to your study. If you are not sure if a list item applies to your research, read the appropriate section before selecting a response.

Materials & experimental systems

n/a	Involved in the study	
[ ]	[x]	Antibodies
[x]	[ ]	Eukaryotic cell lines
[x]	[ ]	Palaeontology
[ ]	[x]	Animals and other organisms
[x]	[ ]	Human research participants
[x]	[ ]	Clinical data

Methods

n/a	Involved in the study	
[x]	[ ]	ChIP-seq
[ ]	[x]	Flow cytometry
[x]	[ ]	MRI-based neuroimaging

Antibodies

Antibodies used	The Ki-67 was utilized to detect cell proliferation with an anti-ki67 (ab15580) (1:200 dilution ratio) and secondary antibody Alexa FluorTM 555 donkey anti-rabbit IgG (A31572) (1:500 dilution ratio).
Validation	Notice that our manuscript involved in validations about these known primary and secondary antibodies with consistent codes.

Animals and other organisms

Policy information about studies involving animals; ARRIVE guidelines recommended for reporting animal research

Laboratory animals	No laboratory animals were used in the study.
Wild animals	The earthworm breed (E. andrei) was bred by Guanglong Earthworm Breeding Institute, Lingshan, Guangxi, China.
Field-collected samples	No field collected samples were used in the study.
Ethics oversight	It did not need ethics oversight because the breeding earthworms often were used for commercial purposes.

Note that full information on the approval of the study protocol must also be provided in the manuscript.

Flow Cytometry

Plots

Confirm that:

- [ ] The axis labels state the marker and fluorochrome used (e.g. CD4-FITC).
- [x] The axis scales are clearly visible. Include numbers along axes only for bottom left plot of group (a 'group' is an analysis of identical markers).
- [x] All plots are contour plots with outliers or pseudocolor plots.
- [x] A numerical value for number of cells or percentage (with statistics) is provided.

Methodology

Sample preparation	Blood cells (domesticated chicken) and somatic cells (earthworm, E. andrei)
Instrument	The genome size estimation was performed using BD LSR Fortessa flow cytometer (BD Biosciences, USA).
Software	Flow cytometry was applied to measure the dna content (genome size) in the earthworm (E. andrei).
Cell population abundance	The cell population abundances have been deposited in Supplementary Figure 2.
Gating strategy	The gating strategy was decided by the empirical balance between relative size of the cells and the granularity (Supplementary Figure 2).

- [x] Tick this box to confirm that a figure exemplifying the gating strategy is provided in the Supplementary Information.
